# Evolutionary and functional analysis of *RBMY1* gene copy number variation on the human Y chromosome

**DOI:** 10.1093/hmg/ddz101

**Published:** 2019-05-21

**Authors:** Wentao Shi, Sandra Louzada, Marina Grigorova, Andrea Massaia, Elena Arciero, Laura Kibena, Xiangyu Jack Ge, Yuan Chen, Qasim Ayub, Olev Poolamets, Chris Tyler-Smith, Margus Punab, Maris Laan, Fengtang Yang, Pille Hallast, Yali Xue

**Affiliations:** 1 Wellcome Genome Campus, Wellcome Sanger Institute, Hinxton, Cambridge CB10 1SA, UK; 2 Department of Genetics, School of Basic Medical Sciences, Tianjin Medical University, Tianjin 300070, China; 3 Institute of Biomedicine and Translational Medicine, University of Tartu, Tartu 50411, Estonia; 4 National Heart and Lung Institute, Imperial College London, London SW7 2AZ, UK; 5 Faculty of Biology, Medicine and Health, School of Biological Science, Division of Musculoskeletal and Dermatological Science, University of Manchester, Manchester M13 9PL, UK; 6 Monash University Malaysia Genomics Facility, Tropical Medicine and Biology Multidisciplinary Platform, Bandar Sunway, Selangor Darul Ehsan 47500, Malaysia; 7 School of Science, Monash University Malaysia, Bandar Sunway, Selangor Darul Ehsan 47500, Malaysia; 8 Andrology Unit, Tartu University Hospital, Tartu 50406, Estonia

## Abstract

Human *RBMY1* genes are located in four variable-sized clusters on the Y chromosome, expressed in male germ cells and possibly associated with sperm motility. We have re-investigated the mutational background and evolutionary history of the *RBMY1* copy number distribution in worldwide samples and its relevance to sperm parameters in an Estonian cohort of idiopathic male factor infertility subjects. We estimated approximate *RBMY1* copy numbers in 1218 1000 Genomes Project phase 3 males from sequencing read-depth, then chose 14 for valid ation by multicolour fibre-FISH. These fibre-FISH samples provided accurate calibration standards for the entire panel and led to detailed insights into population variation and mutational mechanisms. *RBMY1* copy number worldwide ranged from 3 to 13 with a mode of 8. The two larger proximal clusters were the most variable, and additional duplications, deletions and inversions were detected. Placing the copy number estimates onto the published Y-SNP-based phylogeny of the same samples suggested a minimum of 562 mutational changes, translating to a mutation rate of 2.20 × 10^−3^ (95% CI 1.94 × 10^−3^ to 2.48 × 10^−3^) per father-to-son Y-transmission, higher than many short tandem repeat (Y-STRs), and showed no evidence for selection for increased or decreased copy number, but possible copy number stabilizing selection. An analysis of *RBMY1* copy numbers among 376 infertility subjects failed to replicate a previously reported association with sperm motility and showed no significant effect on sperm count and concentration, serum follicle stimulating hormone (FSH), luteinizing hormone (LH) and testosterone levels or testicular and semen volume. These results provide the first in-depth insights into the structural rearrangements underlying *RBMY1* copy number variation across diverse human lineages.

## Introduction

The *RBMY* gene family was first reported in 1993 under the name Y chromosome RNA Recognition Motif as a group of three or more genes located on the long arm of the human Y chromosome, expressed specifically in testis and deleted in two oligospermic individuals (1). It was thus considered a candidate for one of the genes required for spermatogenesis, whose deletion might be a cause of male spermatogenic failure. Subsequent work established that there were six active genes organized into four clusters, as well as five pseudogenes, in the reference sequence (2); confirmed its expression in testis and identified the stages of spermatogenesis at which RBMY protein could be detected (3,4); and investigated its status in men with spermatogenic failure in more detail, showing that all active copies are removed in *AZFb* deletions (5) but not in *AZFc* deletions (6). In addition, several studies delineated its biochemical functions, implicating it in germline-specific aspects of RNA splicing (4,7).

Despite this extensive body of work, major aspects of our understanding of the *RBMY1* genes remained incomplete (8–10). Deletion analyses in humans could not determine whether the spermatogenic failure experienced by *AZFb* deletion carriers was due to loss of *RBMY1* genes or other genes because all three classes of the deletion removed multiple other genes as well (5); no deletions of *RBMY1* genes alone have been reported thus far. Model organism studies have provided only limited insights because of biological differences between species: mice, for example, carry ~50 copies of the homologous gene *Rbmy* and reduction of this number to around four in the Y^d1^ chromosome (complemented by an *Sry* transgene to provide a male phenotype) led to mice with 46% abnormal sperm that were nevertheless fertile (11), contrasting with the human *AZFb* azoospermia phenotype (11). Thus, insights from standard genetic approaches have been limited. Protein analyses have shown that in humans, RBMY protein is present in the midpiece region and in smaller amounts in the tail of mature sperm where there is no transcription, as well as in earlier transcriptionally active cell types, suggesting that RBMY may have additional functions unrelated to transcription; treatment of sperm with antibody directed against an N-terminal epitope of RBMY reduced their motility (3) pointing to one possible additional function.

In parallel, studies of *RBMY1* copy number variation on the human Y chromosome, initially using array CGH (12) and subsequently sequencing (13–16), revealed extensive variation of *RBMY1* copy number in the general population, ranging from 5 to 19, with median (or modal) values of 9, 10.7 and 8.7, respectively. This natural variation in copy number allowed the relationship between *RBMY1* copy number, RBMY protein level and sperm phenotypes to be investigated, leading to a report of positive correlations between copy number, protein level and sperm motility (17). Such an association raises the possibility that sperm competition might lead to positive selection for increased *RBMY1* copy number. Functional links of these kinds would have consequences both for fertility management and treatments (8–10) and for understanding the evolution of the Y chromosome, where interpretations assume neutrality and absence of positive selection based on genetic differences (18).

We therefore set out to reinvestigate the *RBMY1* copy number variation in a worldwide sample of males from phase 3 of the 1000 Genomes Project (19), some 4-fold larger than the combined total used for other samples, and validate some of the copy number measurements by high-resolution fibre-FISH and digital droplet polymerase chain reaction (PCR). With this dataset, and comparisons of available father–son pairs, we estimated the frequency of changes in copy number (i.e. mutation rate) and explored the possibility of positive selection for increasing copy number. Finally, we reinvestigated the relationship between *RBMY1* copy number, sperm motility and other andrological parameters in an independent cohort.

## Results

### Highly variable *RMBY1* clusters from read depth and array CGH analysis in the 1000 Genomes Project samples

**Figure 1:**
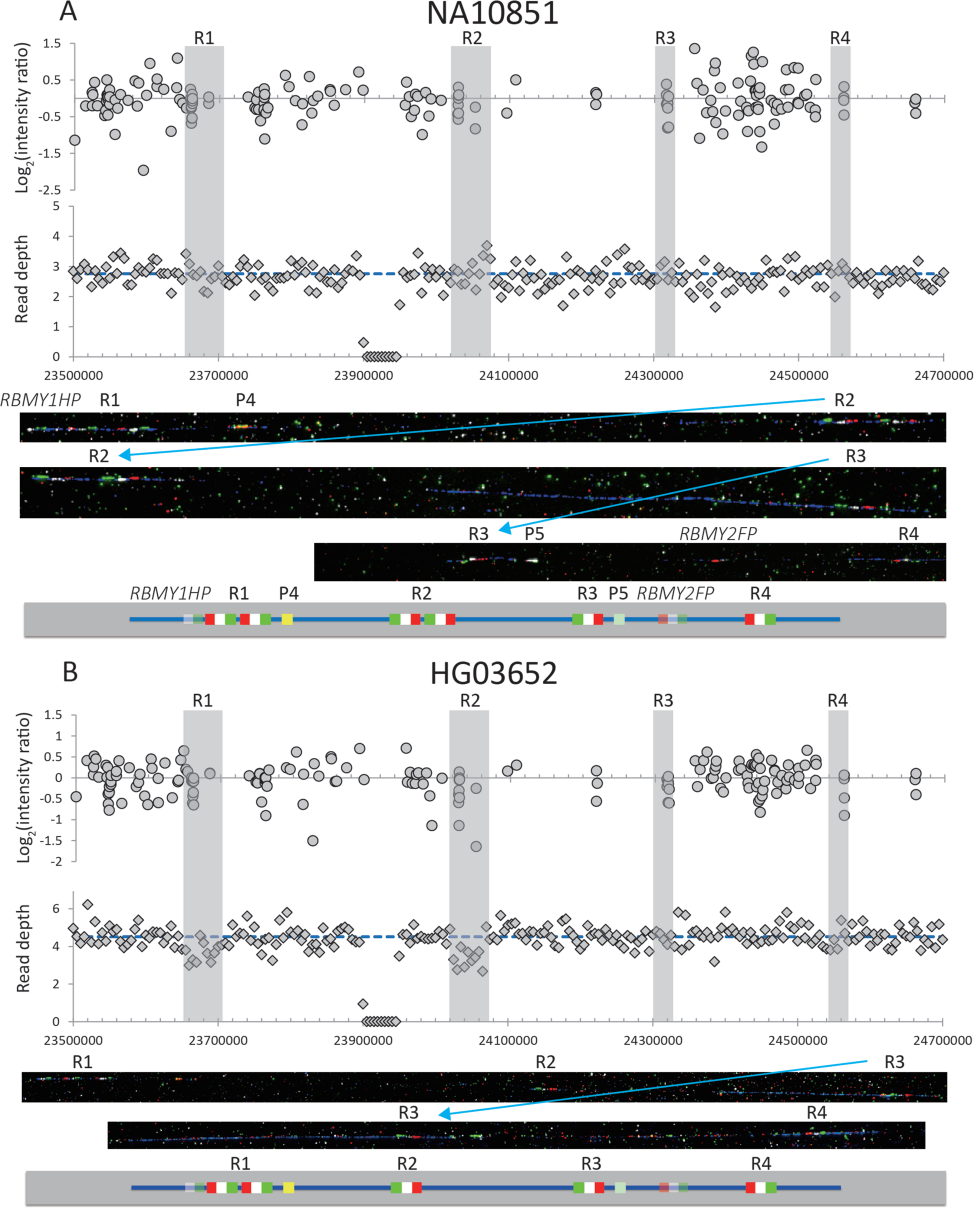
Examples of two samples from the 1000 Genomes Project with different low *RBMY1* copy numbers. (**A**) NA10851 (six copies of *RBMY1*, the same as the reference sequence) and (**B**) HG03652 (five copies). The upper panel shows the log2 ratio intensity plots from array CGH data and the read depth of 5 kb non-overlapping windows from the whole-genome sequencing data. The blue dashed line shows the mean read depth in the unique Y-chromosomal region for each sample. Regions ‘1’–‘4’ are highlighted in grey. Below are fibre-FISH images and a schematic interpretation of the *RBMY1* gene FISH signals: RP11-95B23, blue; P1, red; P2, white; P3, green; P4, yellow; and P5, light green.

**Figure 2:**
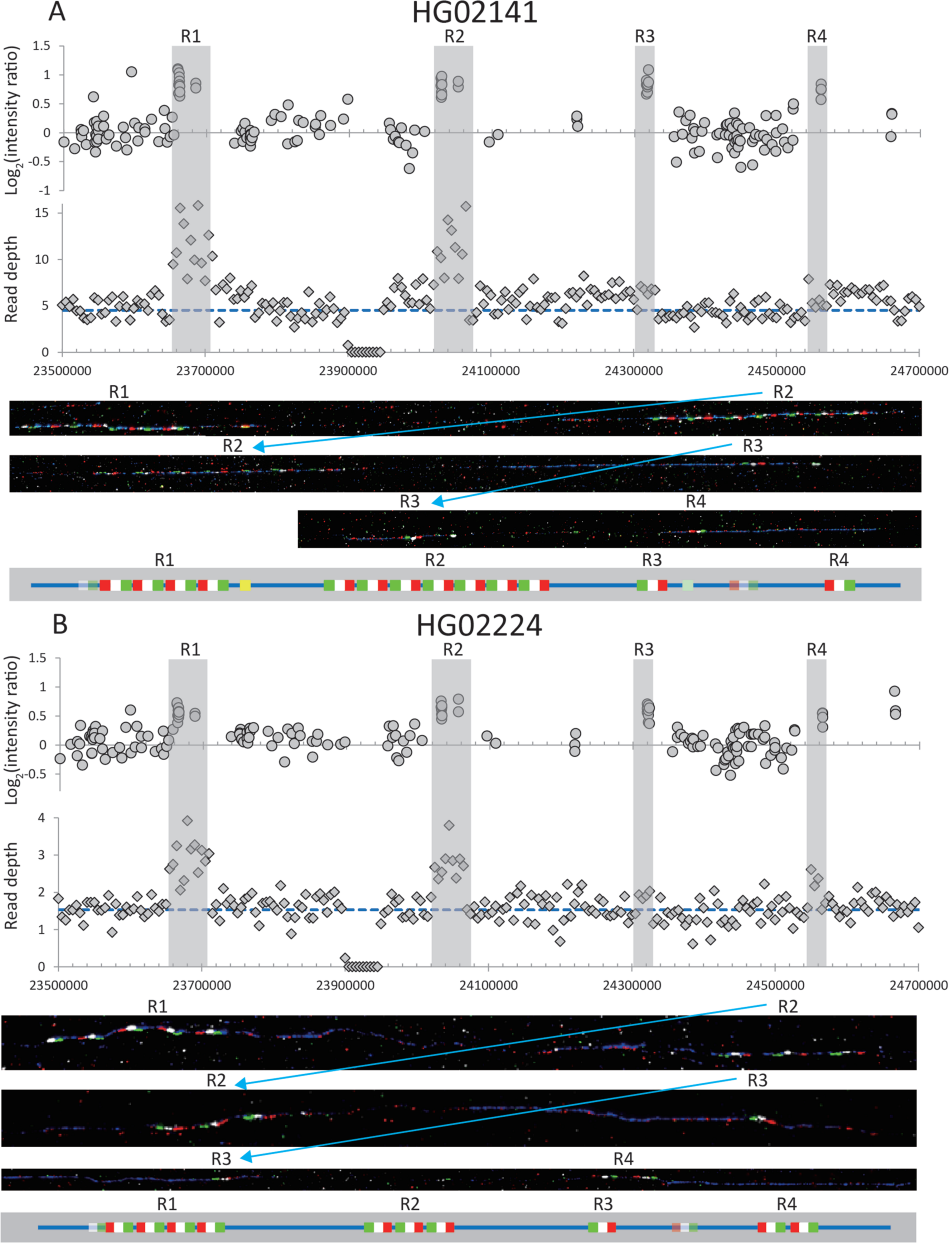
Examples of two samples from the 1000 Genomes Project with different high *RBMY1* copy numbers. (**A**) HG02141 (13 copies) and (**B**) HG02224 (10 copies, including two copies in region ‘4’). The upper panel shows the log2 ratio intensity plots from array CGH data and the read depth of 5 kb non-overlapping windows from the whole-genome sequencing data. The blue dashed line shows the mean read depth in the unique Y-chromosomal region for each sample. Regions ‘1’–‘4’ are highlighted in grey. Below are fibre-FISH images and a schematic interpretation of the *RBMY1* gene FISH signals: RP11-95B23, blue; P1, red; P2, white; P3, green; P4, yellow; and P5, light green.

There are six functional *RBMY1* gene copies with sequence similarity >98% and five pseudogenes in the human Y chromosome reference sequence. The functional copies lie in four separate regions, to which we refer to here as regions ‘1’ (Y:23,673,224–23,711,212), ‘2’ (Y:24,026,223–24,064,214), ‘3’ (Y:24,314,689–24,329,129) and ‘4’ (Y:24,549,583–24,564,028), from proximal to distal (Figs 1 and 2; Supplementary Material, Table S1). The proximal regions ‘1’ and ‘2’ each contain two gene copies in the reference sequence, while the more distal regions ‘3’ and ‘4’ each contain one copy of *RBMY1* (2).

For each of the 1000 Genomes Project male samples (19) we plotted the average read depth in 5 kb windows over a large region of the Y chromosome (Y:22,000,000–29,000,000) and after manual review identified 147 samples with no difference from the reference sequence, 58 ambiguous samples or with more complicated rearrangements and 1017 with departures from the reference read depth. Most variation occurred in the two regions corresponding to *RBMY1* genes: region ‘1’ and region ‘2’ and a few (10 samples) in region ‘4’. No variation was seen in region ‘3’. The pattern of variation was confirmed by manual review of the log2 ratio of array CGH intensity plots (19) from the same samples within the same regions.

We then quantified the variation between samples in an approximate way using the ratio of average read depth across the four regions where the active gene copies are located, compared to the read depth in a nearby single-copy region (see Materials and Methods). Although there are six active copies of *RBMY1* and the five pseudogenes in the reference sequence, the latter are equivalent to just one extra copy in length as the pseudogenes are short and one of them also overlaps with an active gene. Since reads may mis-map to any *RBMY1* copy, we expect a change of ~0.14 in this ratio to be equivalent to a change in gene copy number of one (1/7 = ~ 0.14). These initial ratios ranged from 0.62 to 1.89 suggesting that the copy number ranged between 3 and 12, with a broad distribution across the samples. These findings confirm previous work (12,14–17).

### Detailed structure of the gene cluster revealed by fibre-FISH

Fourteen samples were chosen for detailed analysis by fibre-FISH. A total of 8 of the samples spanned a range of read depths representing almost every copy number from 5 to 13, while the other 6 are 3 pairs of phylogenetically closely related samples forming sister groups that showed different total *RBMY1* copy numbers estimated from the read depth (Table 1). This analysis used three different probes per *RBMY1* gene, which define the gene orientation, plus two further probes: P4 to distinguish the very similar region ‘1’ from ‘2’ and probe P5 to distinguish region ‘3’ from ‘4’. The results confirmed the wide range of copy number variation (5–13) in the *RBMY1* regions of the eight samples as well as the different copy number between three sister pairs of samples in the 1000 Genomes Project phylogenetic tree (19) (Table 1, Figs 1, 2 and 4; Supplementary Material, Figs S1 and S2). The *RBMY1* gene copies are located in four regions as in the human reference sequence, except for one sample (NA19774) where we identified a total of five gene clusters, resulting from a partial duplication of region ‘1’ identified using probe P4 (Fig. 4A). All of the other *RBMY1* copy number changes in these 14 samples occur in proximal regions ‘1’ and ‘2’ except one increase in the region ‘4’ in one sample (HG02224) (Table 1, Fig. 2B). This might be expected since regions ‘1’ and ‘2’ generally have multiple copies of *RMBY1* genes, whereas regions ‘3’ and ‘4’ each generally have only a single copy. The copy numbers vary from 2 to 7 in region ‘1’ and from 1 to 7 in region ‘2’, where two samples (NA19774 and HG03652) have only a single copy of the gene (Figs 1B and 4A).

**Table 1:** *RBMY1* copy number estimates for 14 samples used in fibre-FISH experiments

		**Fibre-FISH**						
**Sample ID**	**Y haplogroup and terminal marker** ^*^	**Region 1**	**Region 2**	**Region 3**	**Region 4**	**Total**	**CN from read depth**	**CN from ddPCR**
HG02141	C3f-F2613	4	7	1	1	13	12.9	12.9
HG01699	E1b1b1b1-L19	6	2	1	1	10	10.7	9.8
HG02224	G2a3-L30	4	3	1	2	10	10.9	10.4
HG02789	G2b1-M283	4	3	1	1	9	9.5	8.2
HG02020	O2a1a-M88	4	5	1	1	11	10.5	10.8
HG02032	O2a1a-M88	4	2	1	1	8	8.4	7.8
HG00704^**^	O3a2b1-M188	5	2	1	1	9	8.7	8.8
HG00692^**^	O3a2b1-M188	3	2	1	1	7	7.0	6.8
NA19774^***^	Q1a2a1-L54	4 + 2	1	1	1	9	8.9	8.5
HG01977	Q1a2a1a1-M3	5	4	1	1	11	10.4	10.7
HG01938	Q1a2a1a1-M3	2	2	1	1	6	6.6	6.3
HG03652	Q1b-F711	2	1	1	1	5	5.1	5.2
HG03899	R1a1a1b2a1-L657	5	4	1	1	11	12.0	10.5
NA10851	R1b1a2a1a2c1i-CTS6581	2	2	1	1	6	5.7	5.8

CN, copy number; ^*^, according to Poznik *et al*. (2016) (19); ^**^, inversion of regions ‘1’ and ‘2’; ^***^, partial duplication of region ‘1’ giving rise to a total of five regions in this sample and inversion of regions ‘3’ and ‘4’.

The fibre-FISH results also showed that different underlying structural changes in the clusters give rise the same total *RBMY1* numbers for four sets of different samples; for example, both HG01699 and HG02224 have a total of 10 gene copies, but the underlying structure of the gene regions is different (Table 1, Fig. 2B; Supplementary Material, Fig. S1A).

We also identified two independent inversion events affecting different regions of the *RBMY1* gene cluster. One is an inversion between regions ‘1’ and ‘2’ where the probe P4 is located upstream of, and close to, region ‘2’ instead of being downstream and close to region ‘1’ as expected (Fig. 4B; Supplementary Material, Fig. S1F). The inversion was found in the two samples HG00704 and HG00692, which are one of the sister pairs in haplogroup O3a2b1-M188, suggesting a single origin for the rearrangement. Another inversion affects regions ‘3’ and ‘4’ inferred from the changed location of probe P5 (Fig. 4A) and was found in one sample, NA19774. This sample shows the highest overall number of rearrangements in the *RBMY1* cluster—it has five gene clusters instead of four, inversion of distal regions ‘3’ and ‘4’ and a deletion with only one *RBMY1* copy remaining in region ‘2’.

Overall, despite the relatively small number of 14 samples included in the fibre-FISH experiments, we have identified an even more extensive variety of rearrangement events in the *RBMY1* genome cluster than initially suggested by read depth-based estimates, including deletions, duplications and inversions.

### *RBMY1* copy number estimates in the 1000 Genomes Project dataset using fibre-FISH standards

The relative total read depths of the four regions combined (compared with an adjacent unique region) were highly correlated with the total number of *RBMY1* genes from the fibre-FISH results of eight samples (y = 0.4945x + 1.1504, R^2^ = 0.97, where x is the read depth ratio and y is the copy number of *RBMY* gene), suggesting that the copy number of *RBMY1* genes can be accurately inferred from this correlation. When the copy numbers of the remaining 6 of the 14 fibre-FISH samples were estimated using this correlation, and compared with the measured fibre-FISH number, 5 of the 6 corresponded precisely, and 1 sample differed by one copy (10 versus 11 copies). Although the number of samples tested is low, these results suggest that copy number estimates are generally reliable but can be slightly less accurate at higher copy numbers.

**Figure 3:**
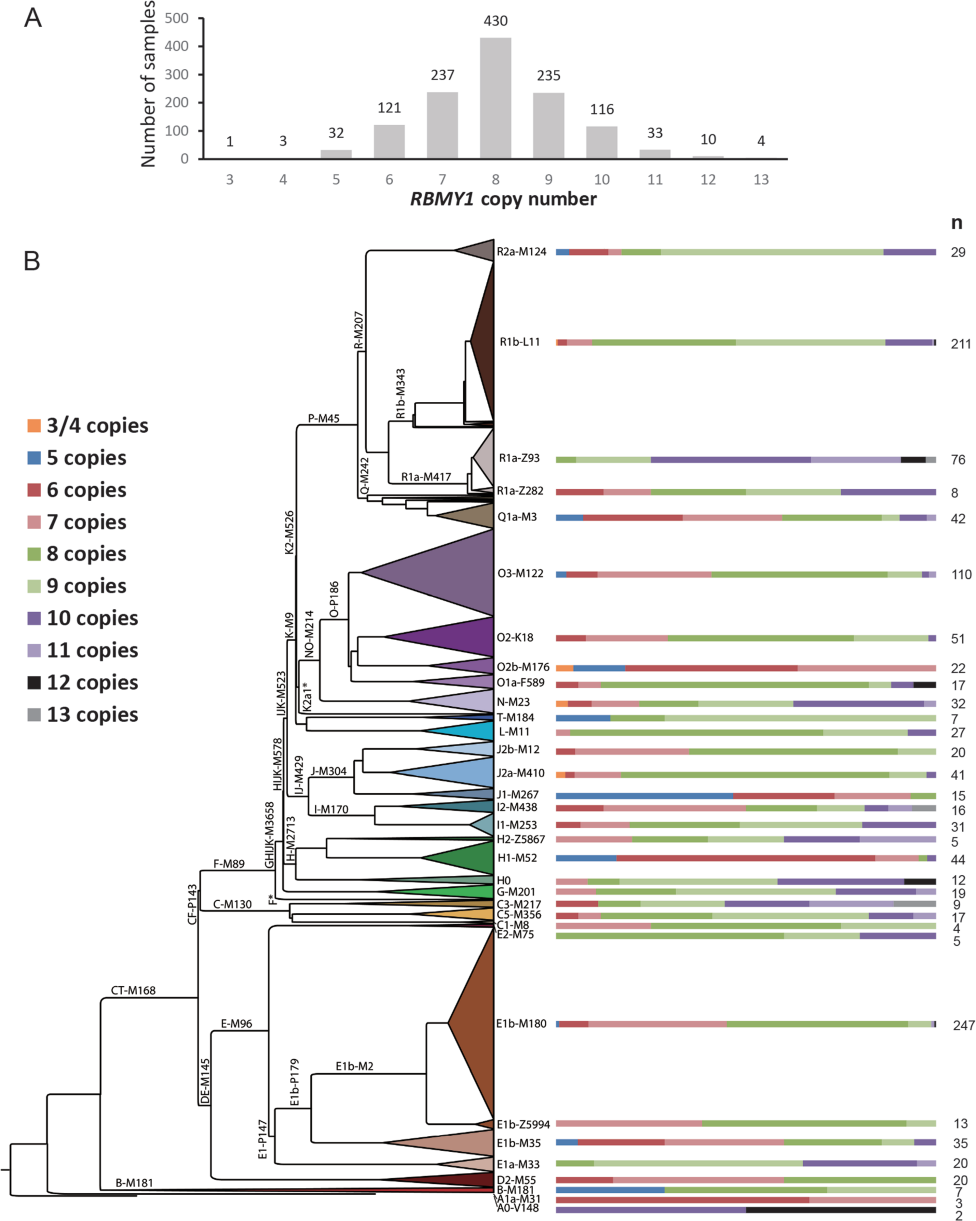
*RBMY1* copy number distribution in the 1000 Genomes Project dataset. (**A**) Copy number distribution across the 1218 samples. (**B**) Copy number distribution across the 1000 Genomes Project Y phylogeny, with the proportion of each copy number in each major clade shown on the right as coloured bars.

*RBMY1* gene copy numbers were then estimated from the read depth for 1218 1000 Genomes Project male samples using the calibration described above. The estimated *RBMY1* copy numbers ranged from 3 to a total of 13 copies (slightly higher than the initial rough estimates), with 8 copies being the most common, found in 430 (35.3%) samples and 7 the second most common, found in 237 (19.5%) (Fig. 3A). It is noteworthy that 6 copies, as in the human reference sequence, was found to be relatively rare and carried only by 121 (9.9%) samples, and only by 6 haplogroup R1b samples (the same haplogroup of most of the human Y reference sequence).

**Figure 4:**
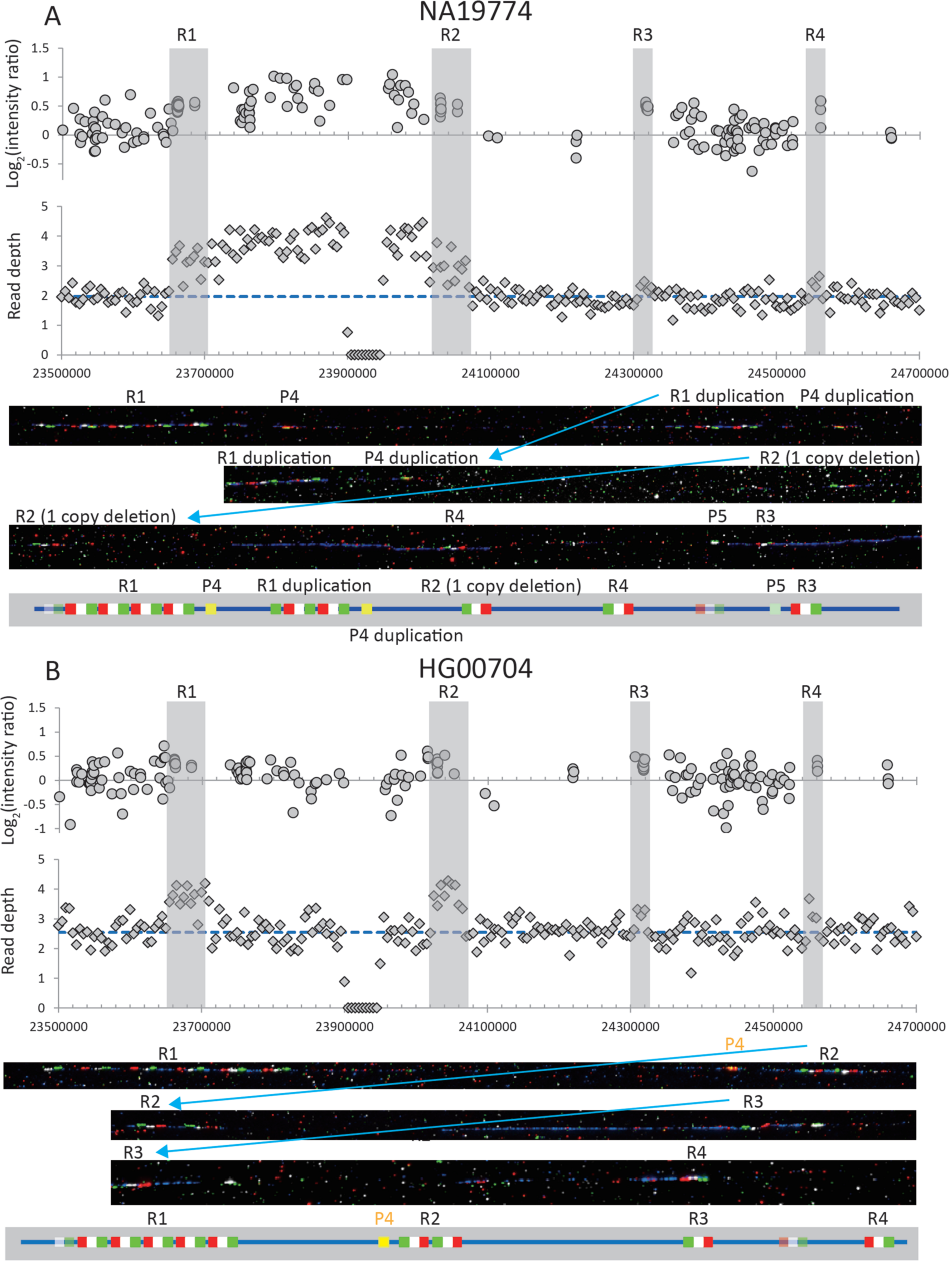
Examples of two samples with rearrangements in *RBMY1* gene cluster: (**A**) NA19774 and (**B**) HG00704. The upper panel shows the log2 ratio intensity plots from array CGH data and the read depth of 5 kb non-overlapping windows from the whole-genome sequencing data. The blue dashed line shows the mean read depth in the unique Y-chromosomal region for each sample. Regions ‘1’–‘4’ are highlighted in grey. Below are fibre-FISH images and schematic interpretation of the *RBMY1* gene FISH signals: RP11-95B23, blue; P1, red; P2, white; P3, green; P4, yellow; and P5, light green.

**Figure 5:**
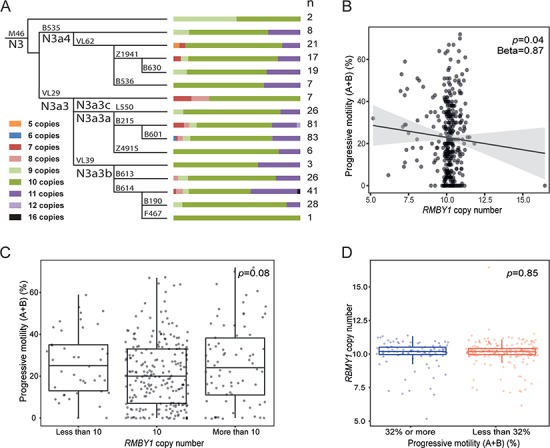
(**A**) Phylogenetic relationships and *RBMY1* copy number distribution among Estonian idiopathic subjects. Coloured bars indicate the proportion of samples carrying each copy number state; n, number of samples. (**B**) Correlation of *RBMY1* copy number and sperm motility in the Estonian cohort. The *P*-value and unstandardized beta from the linear regression test is shown. (**C**) The distribution of progressive sperm motility of the Estonian cohort divided into three groups based on *RBMY1* copy number: less than 10, equal to 10 and more than 10. The *P*-value from the Kruskal–Wallis test is shown. (**D**) The distribution of *RBMY1* copy numbers in Estonian cohort divided into two groups according to the progressive sperm motility: 32% or more and less than 32%. The *P*-value from the Wilcoxon rank sum test is shown.

### *RBMY1* copy number mutation rate estimates

We estimated the mutation rate of *RBMY1* copy number changes by taking advantage of the published robust single-nucleotide variant (SNV)-based Y phylogeny available for the same individuals (Supplementary Material, Fig. S2) (19). Considering the phylogenetic relationships of all the 1218 analysed samples from the 1000 Genomes Project, we counted the minimum number of copy number change events that would explain the current dataset (Supplementary Material, Fig. S2). We counted a total of 562 copy number change events, which corresponds to a rate of 2.20 × 10^−3^ (95% CI: 1.94 × 10^−3^ to 2.48 × 10^−3^) mutations per father-to-son Y transmission (for details, see Materials and Methods). Additionally, we manually checked the average read depth plots and kept only samples where read depth remained constant in the flanking regions around the *RBMY1* genes. For these manually curated 839 samples we counted a total of 371 copy number change events in the phylogenetic tree, which corresponds to a mutation rate of 1.78 × 10^−3^ (95% CI: 1.57 × 10^−3^ to 2.02 × 10^−3^) mutations per father-to-son Y transmission (Supplementary Material, Fig. S2).

Such a high mutation rate of copy number changes is supported by the three pairs of phylogenetically closely related samples (HG02020 and HG02032, HG00704 and HG00692 and HG01977 and HG01938) chosen for fibre-FISH that showed differences in estimated copy numbers from read depth (Table 1; Supplementary Material, Fig. S2). In all cases the estimated copy number changes for these closely related samples were confirmed by fibre-FISH.

Among 60 father–son pairs in the Complete Genomics data also available for the 1000 Genomes Project samples (20), we found one pair showing a copy number increase of ~2 copies between father and son. This mutation of copy number from 10 (father) to 12 (son) was further confirmed using the droplet digital PCR (ddPCR) assays described below. Combining these results with 17 published father–son pairs with no changes (13), we find one mutation event in 77 father-to-son transmissions, corresponding to a mutation rate of 1.3 × 10^−2^ per father-to-son transmission (95% CI: 3.3 × 10^−4^ to 6.8 × 10^−2^). Given the low number of father–son pairs available and the identification of only one mutation, the CI is very wide but we see the measurement as a validation of our estimate from the tree.

### No selection on *RBMY1* copy number changes

It is not possible to apply standard neutrality/selection tests (e.g. 21) for Y-chromosomal copy number variation because of the unique properties of this chromosome, which include a lack of recombination. Therefore, to evaluate whether or not *RBMY1* copy number change is evolving under selection, we developed a different approach that took advantage of the robust phylogeny. Events of copy number increase and decrease for which the direction of change could reliably be inferred were counted in the 1000 Genomes Y phylogeny following the concept of maximum parsimony. We reasoned that if either higher or lower copy number has had a selective advantage in recent human evolution over the 190 000 years covered by this phylogeny, then we should see a bias towards survival of mutations that increase or decrease the copy number, respectively. It was previously reported that increased copy number of *RMBY1* is associated with increased sperm mobility, so we would expect to see more increase events if there is selection for higher motility. To our surprise, however, we observed significantly more decrease events (99 decrease and 77 increase, binomial probability test *P* = 0.015) (Supplementary Material, Fig. S2). As we are working with low-coverage data, we more conservatively only included copy number changes of two or more and also excluded extreme estimates (copy number less than 5 and more than 12). This left a total of 18 copy number increase and 25 decrease events (Supplementary Material, Fig. S2), again more decrease events but no longer statistically significant (Binomial probability test, *P* = 0.07), perhaps due to the low number of total events. We then added increase (6) and decrease (6) events from two additional independent datasets [The Simons Genome Diversity Project (SGDP) (22) and Polaris] (Supplementary Material, Figs S3 and S4C, Table S3), but the combined total was not statistically significant (*P* = 0.06), so there is no evidence for selection for either increased or decreased copy number of the gene. However, the variance of the *RMBY1* copy number in the 1000 Genomes samples (1.65) lies below the 0.5th percentile of the variance distribution of 1000 neutral simulations (95% CI: 6.46 to 66.52) using the same number of mutation events on the same phylogenetic tree; this, in the simplest interpretation, suggests that very low and very high copy numbers of *RMBY1* gene are selected against.

### Establishment and use of ddPCR genotyping

The previous analyses were based on genome sequences, but we wished to extend our work to samples where such sequences were not available. We therefore established two allele-specific ddPCR assays to determine the copy number of functional *RBMY1* genes and to investigate the structural variability of different *RBMY1* gene copies. We chose a T/C paralogous sequence variant that in the human reference sequence distinguishes the four proximal gene copies in regions ‘1’ and ‘2’, called the ‘T-assay’ from the two distal copies in regions ‘3’ and ‘4’, called the ‘C-assay’. The total copy numbers from T- and C-assays are highly correlated with the measurements from the 14 fibre-FISH experiments (‘gold standards’) (y = 0.9582x + 0.1943, R^2^ = 0.98), and the difference between the two methods was less than 0.8 copies (Table 1).

We next typed 376 Estonian idiopathic infertility subjects carrying Y lineage N3-M46, one of the most frequent Y lineages in Estonia, present in ~31% of both the general population (23) and the idiopathic infertility subjects in our cohort, using both ddPCR T- and C-assays. Most of the variation we see in the *RBMY1* copy number estimates comes from the T-assay, specific to regions ‘1’ and ‘2’, ranging from 3 to 14 copies, with 8 being the most common in this cohort. In contrast, very little variation in the copy numbers was found based on the C-assay, specific to regions ‘3’ and ‘4’, with the majority (370 out of 376) of samples carrying two copies of *RBMY1*.

Five samples in the cohort with three copies of *RBMY1* estimated from the C-assay shared a sub-lineage of N3-M46 (N3a3a-L550), suggesting a single rearrangement event, potentially resulting from a gene duplication. Such an example is seen in HG02224, carrying three copies according to the C-assay and a total of three gene copies in regions ‘3’ and ‘4’ according to fibre-FISH (Table 1). A sixth sample with four copies based on C-assay could be due to gene conversion, as again seen in sample of HG01699 (three copies estimated based on C-assay but a total of two genes in regions ‘3’ and ‘4’ according to fibre-FISH). Unfortunately, suitable material for fibre-FISH was not available from the Estonian samples to test these hypotheses.

Overall, the total *RBMY1* copy number (sum of T- and C-assays) among the Estonian subjects ranged from 5 to as high as 16, with a mode of 10 (247 or 65.7% of samples), showing high levels of variation across the haplogroup N3a phylogenetic tree (Fig. 5A; Supplementary Material, Fig. S4A), as also seen in the 1000 Genomes Project samples.

### Association of *RBMY1* copy number with sperm parameters in an Estonian cohort

Among 376 Estonian subjects diagnosed with idiopathic male factor infertility (Table 2), the estimated copy number of the *RBMY1* genes showed no significant associations with sperm parameters (concentration, total count and progressive motility), hormonal levels (total testosterone, serum LH and FSH) and testicular and semen volume (linear regression analysis; Fig. 5B; Supplementary Material, Fig. S5). We did not find an association between copy number of the *RBMY1* genes and progressive motility using only a subset of the samples from the moderate oligozoospermic subjects (*n* = 216) either, although the reduction in sperm production might in principle cause a secondary decrease in sperm motility (Supplementary Material, Fig. S6).

**Table 2:** Characteristics of the Estonian idiopathic infertility cohort (*n* = 376)

**Parameter**	**Mean (±SD)**	**Median (5** ^**th**^ **–95** ^**th**^ **percentile)**
Age (years)	33.9 (8.1)	32.4 (23.2–48.9)
BMI (kg/m^2^)	26.6 (4.6)	26.1 (20.3–35.5)
Abstinence period (days)	3.88 (2.74)	3.00 (2.00–7.00)
Total testis volume (ml)	37.9 (9.08)	38.0 (21.0–50.0)
Semen volume (ml)	3.65 (1.73)	3.50 (1.30–7.20)
Sperm concentration (million/ml)	4.89 (4.77)	4.00 (0–14.00)
Total sperm count (million/ejaculate)	14.8 (11.8)	13.0 (0–36.0)
Progressive motility (A + B) (%)	22.8 (16.6)	21.0 (0–52.0)
FSH (IU/L)	9.29 (7.76)	6.71 (1.90–25.7)
LH (IU(L)	5.08 (2.52)	4.56 (2.00–9.82)
Total testosterone (nmol/L)	17.6 (6.42)	17.0 (8.65–29.4)

We also failed to replicate the reported association between *RBMY1* copy number and progressive sperm motility (17) using two further approaches. Firstly, no significant difference of progressive sperm motility was detected among infertile groups with different pooled *RBMY1* copy numbers: less than 10 (*n* = 41), equal to 10 (*n* = 229) and more than 10 (*n* = 76) (*P* = 0.08, Kruskal–Wallis test) (Fig. 5C). Secondly, subjects with reduced (<32%, *n* = 246) and normal (≥32%, *n* = 101) progressive motility did not differ for their *RBMY1* copy number distribution (*P* = 0.85, Wilcoxon rank sum test) (Fig. 5D). Azoospermic and other subjects who lacked the sperm motility information were excluded from both analyses.

Taken together, we find that *RBMY1* copy number was not associated with any of the tested parameters in the Estonian idiopathic infertility cohort analysed.

## Discussion

In this study, we have investigated the variation in the structure and copy number of the *RBMY1* gene family within the general population, how this arises by mutation, how it may be shaped by selection, and, by studying subjects with unexplained spermatogenic failure, how it may influence sperm phenotypes. We now discuss each of these topics.

The high level of variation in *RBMY1* copy number (second on the Y chromosome only to *TSPY* (24)) has been well established by previous studies in several populations such as the UK (12), Denmark (13), China (17) and worldwide samples (14,15) including those from the 1000 Genomes Project (16,19). The data were generated using diverse techniques: array CGH (12,19), sequencing (14–16,19) or ddPCR (17), and thus this conclusion, confirmed by the present work, is well established. Nevertheless, the numbers of copies differ between studies, and it is unclear how much of this variation is biological and how much due to technical differences. In addition, the variability is distributed over several gene clusters and contains complexities beyond simple variation in copy number. How much variation is there in the number of clusters and in their individual structures and sequences? *RBMY1* copies in regions ‘1’ and ‘2’ are >99.89% identical to one another in the reference sequence, and in regions ‘3’ and ‘4’ are 99.92% identical, but regions ‘1’ plus ‘2’ are only <99.39% identical to regions ‘3’ plus ‘4’. Currently, even *de novo* assembly (13,25) has not been successful in determining the details of such complex structures. Thus, multicolour fibre-FISH, which provides an independent gold standard approach to determining the absolute copy number and structural organization, was a key component of our study. It provided accurate copy number standards for calibration of the entire dataset using samples that are available to all for future work (20). Beyond this, it revealed gross structural variation: the existence of a fifth gene cluster in one sample and two independent inversion events between regions ‘1’ and ‘2’ and between regions ‘3’ and ‘4’, respectively. Within the four gene clusters present in 13 of the 14 samples, most copy number variation occurred in regions ‘1’ and ‘2’, which usually each have multiple *RBMY1* copies. This higher level of variation associated with multiple copies is expected, since duplication or deletion of tandem copies in misaligned sister chromatids is more frequent than duplication or deletion of a single copy (26). Nevertheless, two *RBMY1* copies were present in region ‘4’ in one sample, with a similar spacing (at fibre-FISH resolution) to the genes in the other clusters, perhaps as a result of gene conversion from region ‘1’ or ‘2’ or recurrence of the initial duplication event. While a complete understanding of the complexities of this gene family will require future improved *de novo* assemblies using accurate long reads, we already see from the fibre-FISH results how frequent increase or decrease in copy number within a tandem cluster, moderately frequent inversion events between adjacent pairs of clusters and possibly less frequent transfer of information between more distant clusters can explain the patterns of sequence identity observed in the reference sequence and the variation in the population.

High levels of variation in the population imply a high mutation rate. We assessed this rate using two approaches. The most direct was to compare 60 father–son pairs using available sequence data from the 1000 Genomes Project (20), where we detected one mutation. Combined with the report of zero changes in 17 Danish father–son pairs, this provides a mutation rate of 1 in 77 transmissions or 1.3 × 10^−2^ per father-to-son transmission, with very wide confidence intervals (95% CI: 3.3 × 10^−4^ to 6.8 × 10^−2^). Our second approach was to take advantage of the simple inheritance pattern of the Y chromosome and count the copy number changes in the Y phylogenetic tree, leading to a rate of 2.20 × 10^−3^ (95% CI: 1.94 × 10^−3^ to 2.48 × 10^−3^) mutations per father-to-son transmission. This estimate has not been corrected for recurrent mutations that are likely to have occurred on long branches, so is a minimum estimate, but even so reveals a high rate comparable to Y-STRs (27,28). One factor we are unable to correct for, but which might contribute to the high estimated mutation rate, is the source of DNA for the 1000 Genomes Project samples. Many of these originate from cell lines and may therefore contain *in vitro* rearrangements. However, we expect this not to influence our calculations substantially for three reasons. Firstly, among 77 father–son pairs we also identify one instance of *RBMY1* copy number change. Secondly, it is highly likely that our copy number change count is an underestimate due to recurrent events, and we count any change as one event. Thirdly, a high mutation rate is also supported by the high levels of *RBMY1* copy number variation seen within the Estonian haplogroup N3a phylogenetic tree based on blood DNA (Fig. 5A). We were unable to calculate a mutation rate for these samples using the above-mentioned approach, but copy number changes were found in all the N3a sublineages that have a time to most common recent ancestor of only 5.0 kya (CI: 4.4 to 5.7 kya) (23).

The presence of multiple very similar copies of *RBMY1* on the Y chromosome—unusual for a protein-coding gene—together with specific suggestions that RBMY might influence sperm motility (3,17) led to the question of whether natural selection might be influencing *RBMY1* copy number in the population. We therefore again took advantage of the Y phylogenetic tree to search for evidence of selection for increased or decreased copy number over the 190 000 years of evolution recorded by the tree. We did not find any evidence for selection for increased copy number; on the contrary, we see a slightly higher number of decrease events, but not one that is statistically significant. We found likely purifying selection against very low or high numbers of *RBMY1* genes. This conclusion of a lack of positive selection for copy number change fits well with the lack of association of copy number with sperm mobility (Fig. 5 and below), while the selection to maintain number of *RMBY1* genes within a certain range confirms their functional importance.

The third area that we investigated was to search directly for an association between *RBMY1* copy number and sperm motility, as well as other sperm parameters. We found no evidence for such an association, contrasting with the previous work (17). There could be a number of reasons for this difference. Firstly, there are no clearly and exclusively objective criteria for the quantitative measurement of sperm motility (e.g. 29) apart from rare cases with mostly immotile sperm. Thus, the criteria applied in different centres may differ. Secondly, there could be biological differences between populations, as demonstrated for other Y-linked phenotypes where, for example, the *gr/gr* and *b2/b3* deletions are well-documented risk factors for spermatogenic impairment in some populations but not in others (8). In these cases, the haplogroup background of the deletion is the key factor and differs between populations. The sperm motility measurements were made from carriers of different haplogroups: O3 (17) or N (this work), so this difference should therefore be considered. Thirdly, errors in *RBMY1* copy number estimation might underlie the association differences. We believe that the calibration of our estimates using fibre-FISH, together with the correspondence of our population frequency distributions, which have modal values of 8, to those reported by others (13–16), provide strong support for our estimates. Also, both the read depth approach used on the 1000 Genomes Project data and ddPCR assays used for Estonian idiopathic subjects found 10 copies to be the most frequent state in haplogroup N3. In contrast, Yan *et al*. (17) found distributions with modal values of 6, both in haplogroup O3 and in other haplogroups. O3 in our data shows a modal value of 8 (Supplementary Material, Fig. S4B). We therefore suggest that these copy number estimates and the reported association need to be confirmed.

We conclude that the *RBMY1* gene family shows some of the highest levels of variation in copy number and structural organization yet reported for any human gene family and that current techniques allow this variation to be characterized in detail. The high variability is a consequence of both high levels of increase and decrease in tandem clusters and additional gross rearrangements including inversions. However, no functional consequences of this variation were detected on a broad range of analysed male reproductive parameters and infertility in this study.

## Materials and Methods

### Read depth analyses for the 1000 Genomes Illumina data and Complete Genomics data

We re-analysed here the Y-chromosomal sequence data of 1244 males from the phase 3 of 1000 Genomes Project generated by whole-genome sequencing (19). The average of read depth of 5 kb non-overlapping windows over a large region of the Y chromosome (Y:22,000,000–29,000,000) were calculated, plotted and manually checked twice. The ratio of average read depth for two sets of regions were then calculated from the BAM files of the 1000 Genomes phase 3 male samples. The first set of regions consisted of the four segments (‘1’ Y:23,673,224–23,711,212; ‘2’ Y:24,026,223–24,064,214; ‘3’ Y:24,314,689–24,329,129 and ‘4’ Y:24,549,583–24,564,028) where the six active *RBMY1* genes are located. The second region was Y:23,255,000–23,555,000, a unique region downstream of the *RBMY1* gene cluster. Among these samples, 22 failed our analysis pipeline. Additionally, four samples (HG02070, HG03077, HG03352 and HG03812) that showed duplication of the whole *AZF* region (19) were excluded, leaving a total of 1218 samples with *RBMY1* copy number estimates (Supplementary Material, Table S2).

The same ratios were also calculated for the 60 father–son pairs from the 1000 Genomes Project phase 3 Complete Genomics data from the available read depth at each position, e.g. ftp://ftp-trace.ncbi.nih.gov/1000genomes/ftp/phase3/data/NA12750/cg_data/REF_LCL/coverageRefScore-chrY-GS0000164115-ASM.tsv.bz2 We used the gcCorrectedCoverage column in the file.

### Array CGH analysis of the 1000 Genomes samples

The array CGH data and generation of log2 intensity ratios have previously been described (19). We extracted these ratios for genomic positions between Y:24,000,000–28,000,000 (GRCh37) for all of the 1000 Genomes Project male samples, plotted them using R and viewed them manually.

### Molecular combing fibre-FISH

The cell lines for 14 samples (HG03652, NA10851, HG01938, HG02032, HG00692, NA19774, HG00704, HG02789, HG01699, HG02224, HG01977, HG02020, HG03899 and HG02141) chosen for fibre-FISH experiments from the 1000 Genomes Project were purchased from NHGRI Sample Repository for Human Genetic Research at the Coriell Institute for Medical Research.

In total, five PCR probes were chosen, and their primers were designed using Primer3 (v4.1.0) (Supplementary Material, Table S4). The *RBMY1* genes (~14 kb in length) were covered by three PCR products (probe P1: 4.3 kb, probe P2: 3.5 kb and probe P3: 4.0 kb long). Two other probes were designed to hybridize outside of the *RBMY1* genes: probe P4 to around 42 kb downstream of region ‘1’ to distinguish regions ‘1’ and ‘2’ and probe P5 ~27 kb downstream of region ‘3’ to distinguish regions ‘3’ and ‘4’. The BAC clone RP11-95B23 was used to highlight the target region. Briefly, the BAC DNA and the PCR products were amplified and labelled with GenomePlex Whole Genome Amplification Kits (Sigma-Aldrich, Haverhill, UK) as described in (19). RP11-95B23 was labelled with biotin 16-dUTP; P1 with digoxigenin 11-dUTP; P2 with Cy5xx-dUTP; P3 with DNP 11-dUTP; P4 with digoxigenin 11-dUTP and DNP 11-dUTP; and P5 with DNP 11-dUTP and Cy5xx-dUTP (all from Jena Bioscience, Jena, Germany). Molecular combing fibres preparation and fibre-FISH experiments were carried out as described previously (30). The order of the probes on single-molecule DNA fibres allowed us to determine both the copy number and orientation of *RBMY1* genes and neighbouring genomic regions and to identify other structural changes, for example, inversions.

### Copy number estimates of *RMBY1* from read depth calibrated by fibre-FISH

The linear correlation between (x) the sum of read depth ratios of the four *RBMY1* regions and (y) the accurate copy numbers of *RBMY1* genes from fibre-FISH were calculated in Excel. We used 8 out of 14 samples with accurately measured copy number from the fibre-FISH experiments, including the sample with 2 copies of *RBMY1* in region 4, for the initial correlation calculation, and the remaining 6 to test the accuracy of the inferred copy numbers using the correlation formula.

### Estimation of copy number mutation rate in the 1000 Genomes data

The published detailed phylogenetic tree (19) was used to count the minimum number of copy number changes that would explain the observed genotype patterns in the 1000 Genomes Project samples. For major branches of the tree we assumed that the most common copy number state among its members also corresponds to the ancestral copy number state in this subgroup of Y chromosomes. Any copy number change in the phylogenetic tree was counted as one event even if a difference of multiple copies was observed. The number of *RBMY1* copy number change events among all 1218 samples analysed was 562, and there were 371 events among the manually curated set of 839 samples.

A similar approach to Teitz *et al*. 2018 (16) was used to estimate the mutation rate for *RBMY1* copy number changes. The published tree contains a total of 60 555 bi-allelic SNVs derived from 1244 males across 10.3 Mb of accessible DNA (19). Removal of 471 singletons specific to 26 samples excluded from the current analysis left a total of 60 084 SNVs for the 1218 samples. For the manually curated set of 839 samples another 11 184 singletons were removed, leaving 48 900 SNVs. A Y-chromosomal mutation rate of 0.76 × 10^−9^ (95% CI: 0.67 × 10^−9^ to 0.86 × 10^−9^) SNV mutations per bp per year was used (31). A total of 127.7 years per SNV mutation was then calculated (0.76 × 10^−9^ × 10.3 × 10^7^)^−1^. This was converted into generations assuming a 30 year generation time (32). Each SNV thus corresponds to 4.26 generations, translating into a total branch length of 255 850 generations for 1218 samples and 208 226 for the manually curated 839 samples and yielding a rate of 2.20 × 10^−3^ (95% CI: 1.94 × 10^−3^ to 2.48 × 10^−3^) and 1.78 × 10^−3^ (95% CI: 1.57 × 10^−3^ to 2.02 × 10^−3^) mutations per father-to-son Y transmission, respectively. The confidence interval of the SNV mutation rate was used to obtain the confidence interval for the copy number change mutation rate.

### Simulations of *RBMY1* copy number changes

First, we identified confident mutation changes from our real data by counting the ones on the phylogeny where the ancestral state was clear (176 events) and then scaled them up to the total number of mutations (562 events). In this way we inferred ~227, 54, 16, 13 and 3 events for 1, 2, 3, 4 and 5 copy number decreases, compared with 185, 42, 19 and 4 for corresponding increases. These changes were then randomly assigned on the branches of the same phylogenetic tree according to the branch length in each of the 1000 simulations (i.e. the longer the branch, the higher probability to be assigned a change). We assumed the most common copy number of *RBMY1* genes of eight as the ancestral state. We then determined the final copy number of *RBMY1* genes at each tip of the tree taking into account the changes in the each simulation, and we treated negative copy numbers as zero. Finally, we calculated the variance of the *RBMY1* copy numbers from each of the 1000 simulations and compared this with the empirical variance of *RBMY1* copy number changes. The simulation was done using a custom R script.

### *RBMY1* copy number estimation and Y phylogenetic tree construction for the SGDP, the HGDP and Polaris datasets

The SGDP (22), the HGDP (33) and Polaris (https://github.com/Illumina/Polaris) high-coverage whole-genome sequence data were mapped to GRCh38 and processed using the standard WSI pipelines. Initially, samples overlapping with the 1000 Genomes Project dataset were excluded, leaving a total of 189 unique samples. A total of 16 samples overlapping with the 1000 Genomes Project were included as internal controls. Thus, a total of 205 samples were used for downstream analysis. The *RBMY1* copy numbers for these samples were estimated as described above for the 1000 Genomes Project data.

The genotypes of the 10.3 Mb of chromosome Y sequence previously defined as accessible to short-read sequencing (34) were jointly called using bcftools (v1.8) with minimum base quality 20, mapping quality 20 and defining ploidy as 1. The calls were then filtered as follows: removing SNVs within 5 bp of an indel (SnpGap), removing indels and calls with read depth less than 3. If multiple alleles were supported by reads, then the fraction of reads supporting the called allele should be ≥0.8. Additionally, any site with ≥10% missing calls was removed. We constructed a maximum likelihood Y phylogeny using RAxML v8.2.10 with the GTRGAMMA substitution model using a final set of 33 796 SNVs (35). The tree was visualized using the FigTree software (http://tree.bio.ed.ac.uk/software/figtree/) with midpoint rooting.

### Estonian idiopathic azoo- and oligozoospermic cohort

The study was approved by the Ethics Review Committee on Human Research of the University of Tartu, Estonia (152/4, 221/M-5, 272/M-13) and genotyping at the Wellcome Sanger Institute under WTSI HMDMC 17/105. Informed consent was obtained from all subjects included in the study. The study subjects were collected at The Andrology Centre at Tartu University Hospital during the period from 2003 to 2015. The collection of samples, definition of causal factors for severe male factor infertility and idiopathic infertility, semen analysis and hormone assays have previously been described in detail (36). Briefly, the study group of severe male factor infertility was formed based on reduced spermatozoa count (<39 million per ejaculate) in at least two consecutive semen analyses (37). Men with known potential cause of male factor infertility were excluded, including cryptorchidism, testicular cancer, orchitis/epididymitis, mumps orchitis, varicocele, testis trauma and any subject with large chromosomal abnormalities and Y-chromosomal microdeletions (*AZFa*, *AZFb*, *AZFc*).

In total, 1190 Estonian idiopathic azoo- and oligozoospermic subjects were initially typed for Y-chromosomal *AZFc* partial deletions following a published protocol (38). Among these, 389 subjects carried the *AZFc* partial *b2/b3* deletion known to be fixed in Y haplogroup N (39,40). Due to DNA quantity restrictions, 379 of the samples were selected and typed for Y marker N3-M46 (Tat) by PCR amplifying the underlying region using published primers (41) (Supplementary Material, Table S4) followed by digestion with *Nla*III. The majority of the samples carry the derived allele for N3-M46 with the exception of two samples that were excluded from copy number analysis. Additionally, one sample failed to give reproducible *RBMY1* copy number estimates using the ddPCR assays, leaving a total of 376 samples for downstream analysis (Table 2). According to the severity of spermatogenic impairment, the subjects can be sub-divided as follows: azoospermia, *n* = 27 (spermatozoa missing in the ejaculate); cryptozoospermia, *n* = 26 (spermatozoa count of <1 million/ejaculate); severe oligozoospermia, *n* = 107 (spermatozoa count of 1–10 million/ejaculate) and moderate oligozoospermia, *n* = 216 (spermatozoa count of 10–38 million/ejaculate).

In order to define the haplogroup N3 sublineages these samples carry, we designed PCR primers for 16 additional phylogenetically informative Y markers (23,42) (Supplementary Material, Table S4). The 16 markers were amplified from each sample, pooled in approximately equimolar concentrations, barcoded per sample and sequenced to high coverage (>40×) on Illumina MiSeq using paired-end 250 bp reads. Genotype calling of the marker positions was done using bcftools (v1.8) with minimum base quality 20, mapping quality 20 and defining ploidy as 1. Throughout the paper we follow the established Y nomenclature for haplogroup N (23,42).

### ddPCR genotyping

PCR primers and probes (Supplementary Material, Table S4) for ddPCR assays were designed using Primer3plus (version 2.4.2) according to the recommendations in ddPCR Application Guide (Bio-Rad, Watford, UK). PCR primers for human Y-chromosomal single-copy gene *SRY* used as a reference locus were taken from (43). Two independent allele-specific probes were designed against the single nucleotide variant at position 52 of the *RBMY1* PCR product. One probe is specific to the T-allele (T-assay), which in the human reference sequence (GRCh37) is shared by four *RBMY1* copies in the proximal regions ‘1’ and ‘2’. The second probe is specific to the C-allele (C-assay) shared by two gene copies in the distal regions ‘3’ and ‘4’ in the reference sequence. To increase the specificity of the allele-specific ddPCR assays, unlabelled oligonucleotides identical to the probes were used in both assays (for the T-assay, an oligonucleotide specific to the C-allele was added in equal concentration to the probe, and vice versa) (from Eurogentec Ltd, Seraing, Belgium).

The Bio-Rad QX 200 ddPCR system (44,45) was used to quantify the copy numbers of functional *RBMY1* genes. PCR reactions were prepared according to the manufacturers recommendations, containing the final concentrations of 1X ddPCR Supermix for Probes (no dUTP), 900 nM of PCR primers and 250 nM of probes for both target and reference regions plus 250 nM of unlabelled oligonucleotide and 0.5 U/μL of AluI restriction enzyme. A total of 5 ng of template genomic DNA was used per reaction. The droplet emulsions were prepared by mixing 70 μL of Droplet Generation oil for Probes with 20 μL of PCR reactions using the QX100 Droplet Generator (Bio-Rad). The PCR conditions were the following: for 5 min at 95°C, 40 cycles of 30 s at 94°C and for 1 min at 59.2°C for T-assay or 57.4°C for the C-assay, for 10 min at 10°C and a 4°C hold with ramp rate of 2°C/s. The fluorescence of each droplet was measured using a XQ200 Droplet Reader and QuantaSoft droplet reader software (v1.6.6.0320; Bio-Rad) was used to cluster droplets into distinct fluorescent groups. Copy number was determined by calculating the ratio of target (unknown) and reference concentration.

ddPCR reactions using the T-assay were performed at least twice for every sample. If the difference in copy number estimate between the two replicates was 0.8 or greater, then a third replicate was performed. The copy number estimates from the C-assay were consistently close to two for majority of the samples. Therefore, only samples that differed from two, plus 5% of all other samples, were replicated, each time giving a concordant copy number estimate. The total *RBMY1* copy number is the sum of copy number estimates from the T- and C-assays.

### Statistical analyses

Statistical testing for the genetic associations between *RBMY1* copy number and andrological parameters of 376 Estonian subjects was conducted using RStudio (version 1.1.456) (46). Data were visualized using package ggplot2 (version 3.1.0) (47).

The *RBMY1* copy number, calculated by the QuantaSoft software as the ratio of target and reference concentration (i.e. not binned or rounded), was used as a measure of real copy number in linear regression models. The following parameters were tested for association with *RBMY1* copy number: sperm concentration (million/ml), total sperm count (million/ejaculate), semen volume (ml/ejaculate), total testis volume (ml), progressive motility (WHO motility classes A + B) (%), total testosterone (nmol/l), serum LH (IU/L) and serum FSH (IU/L). The genetic association tests were adjusted for appropriate cofactors; all parameters for age and additionally sperm parameters for abstinence time. For total testosterone level, an additional multiple linear regression analysis was conducted using both age and body mass index (BMI) as cofactors. For all linear regression analyses, natural log transformation was used to achieve an approximately normal distribution of values. Normality of the transformed distribution was assessed visually on histograms and statistically using the Kolmogorov–Smirnov test assuming that under the null hypothesis the observed values are normally distributed (significance level, α = 0.05). In all cases, with the exception of total sperm count, the applied transformation resulted in a close-to-normal distribution.

The non-parametric Wilcoxon rank sum test was used to test for the *RBMY1* copy number distribution difference in subjects with reduced (<32%) and subjects with normal (≥32%) progressive motility and Kruskal–Wallis test for the progressive motility distribution difference in samples with <10, 10 and >10 functional *RBMY1* copies.

In all tests, *P* < 0.05 after adjustment for Bonferroni correction of multiple testing was considered as a statistically significant outcome.

## Supplementary Material

HMG-2019-D-0019_Shi_Suppl_tables_ddz101

HMG-2019-D-0019_Shi_Supplementary_information_figures_ddz101

## References

[1] Ma K., Inglis J.D., Sharkey A., Bickmore W.A., Hill R.E., Prosser E.J., Speed R.M., Thomson E.J., Jobling M., Taylor K. et al. (1993) A Y chromosome gene family with RNA-binding protein homology: candidates for the azoospermia factor AZF controlling human spermatogenesis. Cell, 75, 1287–1295. 8269511 10.1016/0092-8674(93)90616-x

[2] Skaletsky H., Kuroda-Kawaguchi T., Minx P.J., Cordum H.S., Hillier L., Brown L.G., Repping S., Pyntikova T., Ali J., Bieri T. et al. (2003) The male-specific region of the human Y chromosome is a mosaic of discrete sequence classes. Nature, 423, 825–837. 12815422 10.1038/nature01722

[3] Abid S., Sagare-Patil V., Gokral J. and Modi D. (2013) Cellular ontogeny of RBMY during human spermatogenesis and its role in sperm motility. J. Biosci., 38, 85–92. 23385816 10.1007/s12038-012-9281-8

[4] Elliott D.J. (2004) The role of potential splicing factors including RBMY, RBMX, hnRNPG-T and STAR proteins in spermatogenesis. Int. J. Androl., 27, 328–334. 15595951 10.1111/j.1365-2605.2004.00496.x

[5] Repping S., Skaletsky H., Lange J., Silber S., Van Der Veen F., Oates R.D., Page D.C. and Rozen S. (2002) Recombination between palindromes P5 and P1 on the human Y chromosome causes massive deletions and spermatogenic failure. Am. J. Hum. Genet., 71, 906–922. 12297986 10.1086/342928 PMC419997

[6] Kuroda-Kawaguchi T., Skaletsky H., Brown L.G., Minx P.J., Cordum H.S., Waterston R.H., Wilson R.K., Silber S., Oates R., Rozen S. et al. (2001) The *AZFc* region of the Y chromosome features massive palindromes and uniform recurrent deletions in infertile men. Nat. Genet., 29, 279–286. 11687796 10.1038/ng757

[7] Dreumont N., Bourgeois C.F., Lejeune F., Liu Y., Ehrmann I.E., Elliott D.J. and Stevenin J. (2010) Human RBMY regulates germline-specific splicing events by modulating the function of the serine/arginine-rich proteins 9G8 and Tra2-β. J. Cell Sci., 123, 40–50. 20016065 10.1242/jcs.055889

[8] Krausz C. and Casamonti E. (2017) Spermatogenic failure and the Y chromosome. Hum. Genet., 136, 637–655. 28456834 10.1007/s00439-017-1793-8

[9] Colaco S. and Modi D. (2018) Genetics of the human Y chromosome and its association with male infertility. Reprod. Biol. Endocrinol., 16, 14. 29454353 10.1186/s12958-018-0330-5 PMC5816366

[10] Vogt P.H., Bender U., Zimmer J. and Strowitzki T. (2017) In Vogt P.H. (ed), Genetics of Human Infertility. Karger, Basel, Vol. 21, pp. 57–73.

[11] Mahadevaiah S.K., Odorisio T., Elliott D.J., Rattigan A., Szot M., Laval S.H., Washburn L.L., McCarrey J.R., Cattanach B.M., Lovell-Badge R. et al. (1998) Mouse homologues of the human AZF candidate gene *RBM* are expressed in spermatogonia and spermatids, and map to a Y chromosome deletion interval associated with a high incidence of sperm abnormalities. Hum. Mol. Genet., 7, 715–727. 9499427 10.1093/hmg/7.4.715

[12] Wei W., Fitzgerald T.W., Ayub Q., Massaia A., Smith B.H., Dominiczak A.F., Morris A.D., Porteous D.J., Hurles M.E., Tyler-Smith C. et al. (2015) Copy number variation in the human Y chromosome in the UK population. Hum. Genet., 134, 789–800. 25957587 10.1007/s00439-015-1562-5 PMC4460274

[13] Skov L., Danish Pan Genome Consortium and Schierup M.H. (2017) Analysis of 62 hybrid assembled human Y chromosomes exposes rapid structural changes and high rates of gene conversion. PLoS Genet., 13, e1006834. 28846694 10.1371/journal.pgen.1006834 PMC5591018

[14] Ye D., Zaidi A.A., Tomaszkiewicz M., Anthony K., Liebowitz C., DeGiorgio M., Shriver M.D. and Makova K.D. (2018) High levels of copy number variation of ampliconic genes across major human Y haplogroups. Genome Biol. Evol., 10, 1333–1350. 29718380 10.1093/gbe/evy086 PMC6007357

[15] Lucotte E.A., Skov L., Jensen J.M., Macia M.C., Munch K. and Schierup M.H. (2018) Dynamic copy number evolution of X- and Y-linked ampliconic genes in human populations. Genetics, 209, 907–920. 29769284 10.1534/genetics.118.300826 PMC6028258

[16] Teitz L.S., Pyntikova T., Skaletsky H. and Page D.C. (2018) Selection has countered high mutability to preserve the ancestral copy number of Y chromosome amplicons in diverse human lineages. Am. J. Hum. Genet., 103, 261–275. 30075113 10.1016/j.ajhg.2018.07.007 PMC6080837

[17] Yan Y., Yang X., Liu Y., Shen Y., Tu W., Dong Q., Yang D., Ma Y. and Yang Y. (2017) Copy number variation of functional *RBMY1* is associated with sperm motility: an azoospermia factor-linked candidate for asthenozoospermia. Hum. Reprod., 32, 1521–1531. 28498920 10.1093/humrep/dex100

[18] Jobling M.A. and Tyler-Smith C. (2017) Human Y-chromosome variation in the genome-sequencing era. Nat. Rev. Genet., 18, 485–497. 28555659 10.1038/nrg.2017.36

[19] Poznik G.D., Xue Y., Mendez F.L., Willems T.F., Massaia A., Wilson Sayres M.A., Ayub Q., McCarthy S.A., Narechania A., Kashin S. et al. (2016) Punctuated bursts in human male demography inferred from 1,244 worldwide Y-chromosome sequences. Nat. Genet., 48, 593–599. 27111036 10.1038/ng.3559 PMC4884158

[20] The 1000 Genomes Project Consortium (2015) A global reference for human genetic variation. Nature, 526, 68–74. 26432245 10.1038/nature15393 PMC4750478

[21] Sabeti P.C., Schaffner S.F., Fry B., Lohmueller J., Varilly P., Shamovsky O., Palma A., Mikkelsen T.S., Altshuler D. and Lander E.S. (2006) Positive natural selection in the human lineage. Science, 312, 1614–1620. 16778047 10.1126/science.1124309

[22] Mallick S., Li H., Lipson M., Mathieson I., Gymrek M., Racimo F., Zhao M., Chennagiri N., Nordenfelt S., Tandon A. et al. (2016) The Simons genome diversity project: 300 genomes from 142 diverse populations. Nature, 538, 201–206. 27654912 10.1038/nature18964 PMC5161557

[23] Ilumäe A.M., Reidla M., Chukhryaeva M., Jarve M., Post H., Karmin M., Saag L., Agdzhoyan A., Kushniarevich A., Litvinov S. et al. (2016) Human Y chromosome haplogroup N: a non-trivial time-resolved phylogeography that cuts across language families. Am. J. Hum. Genet., 99, 163–173. 27392075 10.1016/j.ajhg.2016.05.025 PMC5005449

[24] Mathias N., Bayes M. and Tyler-Smith C. (1994) Highly informative compound haplotypes for the human Y chromosome. Hum. Mol. Genet., 3, 115–123. 7909247 10.1093/hmg/3.1.115

[25] Kuderna L.F.K., Lizano E., Julia E., Gomez-Garrido J., Serres-Armero A., Kuhlwilm M., Alandes R.A., Alvarez-Estape M., Juan D., Simon H. et al. (2019) Selective single molecule sequencing and assembly of a human Y chromosome of African origin. Nat. Commun., 10, 4. 30602775 10.1038/s41467-018-07885-5 PMC6315018

[26] Carvalho C.M. and Lupski J.R. (2016) Mechanisms underlying structural variant formation in genomic disorders. Nat. Rev. Genet., 17, 224–238. 26924765 10.1038/nrg.2015.25 PMC4827625

[27] Balanovsky O. (2017) Toward a consensus on SNP and STR mutation rates on the human Y-chromosome. Hum. Genet., 136, 575–590. 28455625 10.1007/s00439-017-1805-8

[28] Willems T., Gymrek M., Poznik G.D., Tyler-Smith C., 1000 Genomes Project Chromosome Y Group and Erlich Y. (2016) Population-scale sequencing data enable precise estimates of Y-STR mutation rates. Am. J. Hum. Genet., 98, 919–933. 27126583 10.1016/j.ajhg.2016.04.001 PMC4863667

[29] Talarczyk-Desole J., Berger A., Taszarek-Hauke G., Hauke J., Pawelczyk L. and Jedrzejczak P. (2017) Manual vs. computer-assisted sperm analysis: can CASA replace manual assessment of human semen in clinical practice? Ginekol. Pol., 88, 56–60. 28326513 10.5603/GP.a2017.0012

[30] Algady W., Louzada S., Carpenter D., Brajer P., Farnert A., Rooth I., Ngasala B., Yang F., Shaw M.A. and Hollox E.J. (2018) The malaria-protective human Glycophorin structural variant *DUP4* shows somatic mosaicism and association with hemoglobin levels. Am. J. Hum. Genet., 103, 769–776. 30388403 10.1016/j.ajhg.2018.10.008 PMC6218809

[31] Fu Q., Li H., Moorjani P., Jay F., Slepchenko S.M., Bondarev A.A., Johnson P.L., Aximu-Petri A., Prufer K., de Filippo C. et al. (2014) Genome sequence of a 45,000-year-old modern human from western Siberia. Nature, 514, 445–449. 25341783 10.1038/nature13810 PMC4753769

[32] Fenner J.N. (2005) Cross-cultural estimation of the human generation interval for use in genetics-based population divergence studies. Am. J. Phys. Anthropol., 128, 415–423. 15795887 10.1002/ajpa.20188

[33] Meyer M., Kircher M., Gansauge M.T., Li H., Racimo F., Mallick S., Schraiber J.G., Jay F., Prufer K., de Filippo C. et al. (2012) A high-coverage genome sequence from an archaic Denisovan individual. Science, 338, 222–226. 22936568 10.1126/science.1224344 PMC3617501

[34] Poznik G.D., Henn B.M., Yee M.C., Sliwerska E., Euskirchen G.M., Lin A.A., Snyder M., Quintana-Murci L., Kidd J.M., Underhill P.A. et al. (2013) Sequencing Y chromosomes resolves discrepancy in time to common ancestor of males versus females. Science, 341, 562–565. 23908239 10.1126/science.1237619 PMC4032117

[35] Stamatakis A. (2014) RAxML version 8: a tool for phylogenetic analysis and post-analysis of large phylogenies. Bioinformatics, 30, 1312–1313. 24451623 10.1093/bioinformatics/btu033 PMC3998144

[36] Punab M., Poolamets O., Paju P., Vihljajev V., Pomm K., Ladva R., Korrovits P. and Laan M. (2017) Causes of male infertility: a 9-year prospective monocentre study on 1737 patients with reduced total sperm counts. Hum. Reprod., 32, 18–31. 27864361 10.1093/humrep/dew284 PMC5165077

[37] World Health Organization (2010) WHO Laboratory Manual for the Examination and Processing of Human Semen, 5th edn. World Health Organization, Geneva.

[38] Lin Y.W., Hsu C.L. and Yen P.H. (2006) A two-step protocol for the detection of rearrangements at the AZFc region on the human Y chromosome. Mol. Hum. Reprod., 12, 347–351. 16608905 10.1093/molehr/gal038

[39] Repping S., van Daalen S.K., Korver C.M., Brown L.G., Marszalek J.D., Gianotten J., Oates R.D., Silber S., van der Veen F., Page D.C. et al. (2004) A family of human Y chromosomes has dispersed throughout northern Eurasia despite a 1.8-Mb deletion in the azoospermia factor c region. Genomics, 83, 1046–1052. 15177557 10.1016/j.ygeno.2003.12.018

[40] Fernandes S., Paracchini S., Meyer L.H., Floridia G., Tyler-Smith C. and Vogt P.H. (2004) A large AZFc deletion removes DAZ3/DAZ4 and nearby genes from men in Y haplogroup N. Am. J. Hum. Genet., 74, 180–187. 14639527 10.1086/381132 PMC1181906

[41] Underhill P.A., Shen P., Lin A.A., Jin L., Passarino G., Yang W.H., Kauffman E., Bonne-Tamir B., Bertranpetit J., Francalacci P. et al. (2000) Y chromosome sequence variation and the history of human populations. Nat. Genet., 26, 358–361. 11062480 10.1038/81685

[42] Karmin M., Saag L., Vicente M., Wilson Sayres M.A., Jarve M., Talas U.G., Rootsi S., Ilumae A.M., Magi R., Mitt M. et al. (2015) A recent bottleneck of Y chromosome diversity coincides with a global change in culture. Genome Res., 25, 459–466. 25770088 10.1101/gr.186684.114 PMC4381518

[43] Tomaszkiewicz M., Rangavittal S., Cechova M., Campos Sanchez R., Fescemyer H.W., Harris R., Ye D., O’Brien P.C., Chikhi R., Ryder O.A. et al. (2016) A time- and cost-effective strategy to sequence mammalian Y chromosomes: an application to the *de novo* assembly of gorilla Y. Genome Res., 26, 530–540. 26934921 10.1101/gr.199448.115 PMC4817776

[44] Hindson B.J., Ness K.D., Masquelier D.A., Belgrader P., Heredia N.J., Makarewicz A.J., Bright I.J., Lucero M.Y., Hiddessen A.L., Legler T.C. et al. (2011) High-throughput droplet digital PCR system for absolute quantitation of DNA copy number. Anal. Chem., 83, 8604–8610. 22035192 10.1021/ac202028g PMC3216358

[45] McDermott G.P., Do D., Litterst C.M., Maar D., Hindson C.M., Steenblock E.R., Legler T.C., Jouvenot Y., Marrs S.H., Bemis A. et al. (2013) Multiplexed target detection using DNA-binding dye chemistry in droplet digital PCR. Anal. Chem., 85, 11619–11627. 24180464 10.1021/ac403061n

[46] RStudio Team (2015) RStudio: Integrated Development for R. RStudio, Inc., Boston, MA, http://www.rstudio.com/.

[47] Wickham H. (2016) ggplot2: Elegant Graphics for Data Analysis. Springer, New York.

