# Supplementary material for: Evolutionary and functional analysis of RBMY1 gene copy number variation on the human Y chromosome
Source: Hum Mol Genet. 2019 May 21;28(16):2785–98. doi: 10.1093/hmg/ddz101 (PMC6687947; doi:10.1093/hmg/ddz101)
Supplement: HMG-2019-D-0019_Shi_Supplementary_information_figures_ddz101 [file hmg-2019-d-0019_shi_supplementary_information_figures_ddz101.zip › HMG-2019-D-0019_Shi_Supplementary_information_figures_ddz101.pdf]

Supplemental Information

Evolutionary and functional analysis of *RBMY1* gene copy number variation on the human Y chromosome

Wentao Shi, Sandra Louzada, Marina Grigorova, Andrea Massaia, Elena Arciero, Laura Kibena, Xiangyu Jack Ge, Yuan Chen, Qasim Ayub, Olev Poolamets, Chris Tyler-Smith, Margus Punab, Maris Laan, Fengtang Yang, Pille Hallast and Yali Xue

Supplementary Figures:

Supplementary Figure 1A-J. Detection and validation of *RBMY1* copy numbers for samples from the 1000 Genomes Project. The upper panel shows the log2 ratio intensity plots from array-CGH data and the read depth of 5 kb non-overlapping windows from the whole-genome sequencing data. The blue dashed line shows the mean read depth in the unique Y-chromosomal region for each sample. Regions '1'-'4' are highlighted in grey. Below are fibre-FISH images and schematic interpretation of the *RBMY1* gene FISH signals: RP11-95B23, blue; P1, red; P2, white; P3, green; P4, yellow and P5, light green.

Supplementary Figure 2a-e. The 1000 Genomes Project Y phylogeny, partitioned into subtrees (Supplementary Figure 14 from (1). The *RBMY1* copy number estimate from read depth is shown in the column on the right side of the tree, the left-hand column showing copy number estimates for all 1,218 samples and right-hand column for manually curated set of 839 samples. Samples for which copy number change event was counted are highlighted in light blue. Green and red circles indicate to locations in the tree where a copy number increase or decrease was counted. Two circles indicate the more conservative increase and decrease events where the extreme copy number estimates (less than five and more than 12) were excluded, and a change of at least two copies had occurred.

Supplementary Figure 3. The Y phylogeny of the Simons Genome Diversity Project, the Human Genome Diversity Project and Polaris datasets. The *RBMY1* copy number estimate from read depth is shown in the column on the right side of the tree. Green and red circles indicate to locations in the tree where a copy number increase or decrease event was counted (excluding extreme copy number estimates of less than five and more than 12 and including only changes of at least two copies). Samples overlapping with the 1000 Genomes Project low coverage dataset are shown in blue and were ignored for the increase/decrease counts. The sample name in the tree shows the predicted haplogroup name using the yHaplo software (https://github.com/23andMe/yhaplo), sample ID and country of origin separated by underscores.

Supplementary Figure 4. *RBMY1* copy number distribution among: A. 376 Estonian idiopathic subjects carrying haplogroup N, B. 109 samples from the 1000 Genomes Project dataset carrying haplogroup O3. C. 188 samples from the Simons Genome Diversity Project, the Human Genome Diversity Project and Polaris datasets.

Supplementary Figure 5. Correlation of *RBMY1* copy number with different parameters in the Estonian subjects cohort. The *p*-value and unstandardized beta from the linear regression test is shown for each parameter.

Supplementary Figure 6. Correlation of *RBMY1* copy number with progressive sperm motility (A+B) in the Estonian patients with moderate oligozoospermia (n=216). The *p*-value and unstandardized beta from the linear regression test is shown.

Supplementary tables:

Table are provided as an Excel file.

Supplementary table 1. Classification of four gene cluster regions.

Supplementary table 2. *RBMY1* copy number estimates from read depth for 1,218 1000 Genomes Project males.

Supplementary table 3. *RBMY1* copy number estimates from read depth for SGDP, HGDP and Polaris datasets.

Supplementary table 4. PCR primers and probes used.

Supplemental references:

1. Poznik, G.D., Xue, Y., Mendez, F.L., Willems, T.F., Massaia, A., Wilson Sayres, M.A., Ayub, Q., McCarthy, S.A., Narechania, A., Kashin, S. *et al.* (2016) Punctuated bursts in human male demography inferred from 1,244 worldwide Y-chromosome sequences. *Nat Genet*, **48**, 593-599.

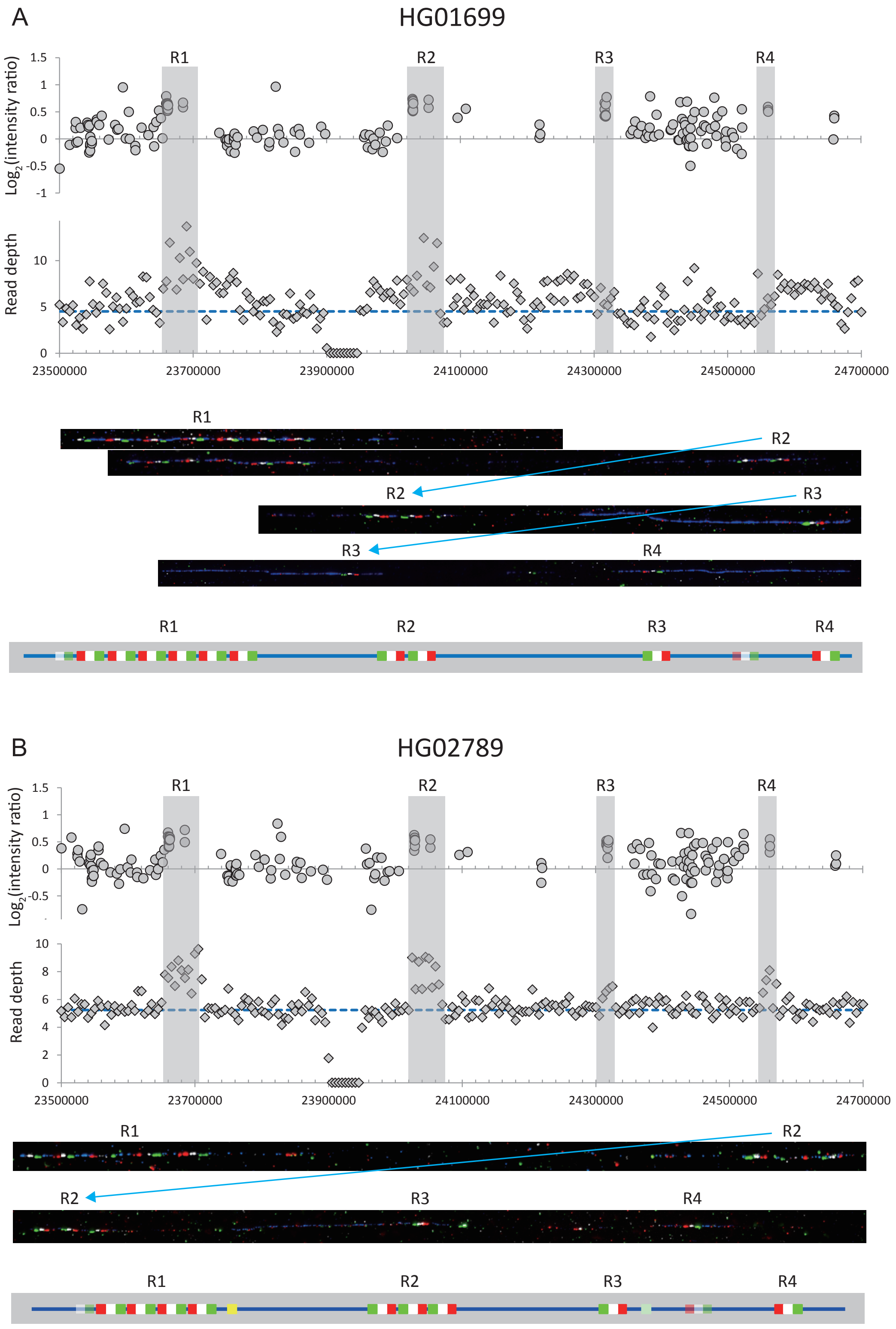

Shi et al Supplementary Figure 1

C

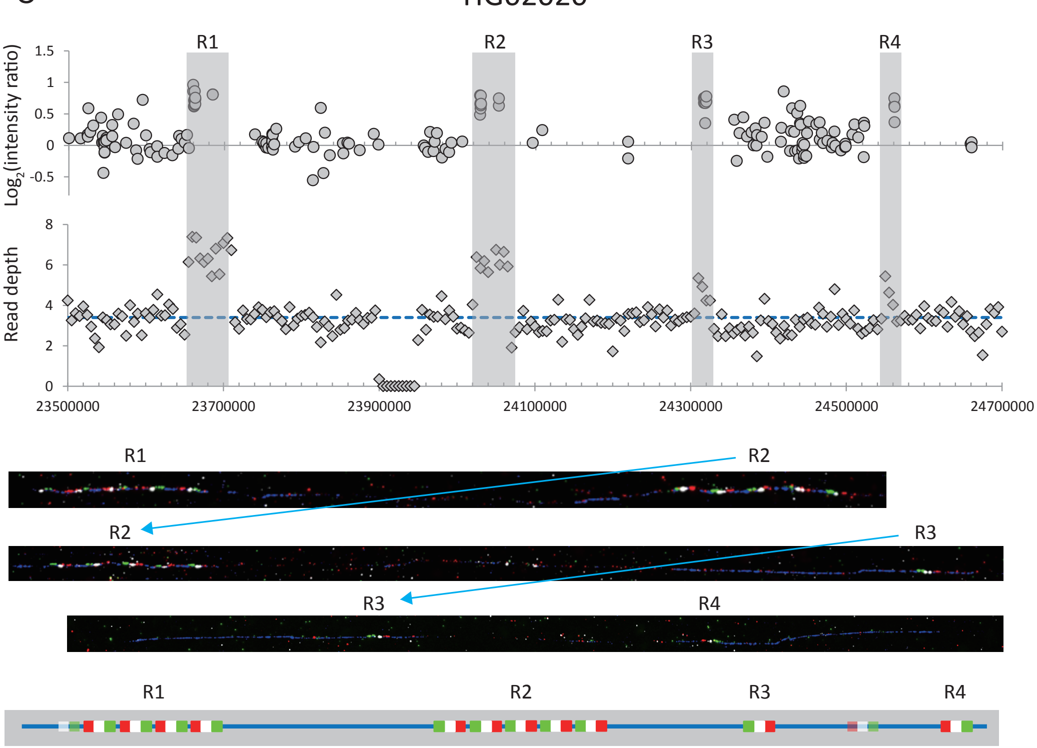

D

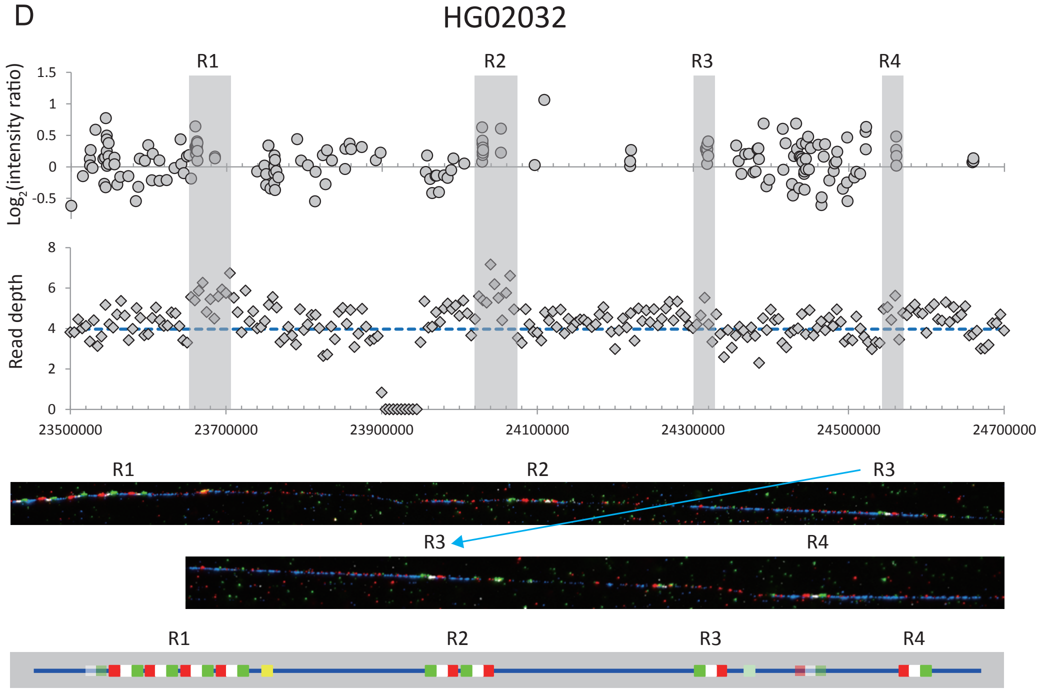

Shi et al Supplementary Figure 1

E

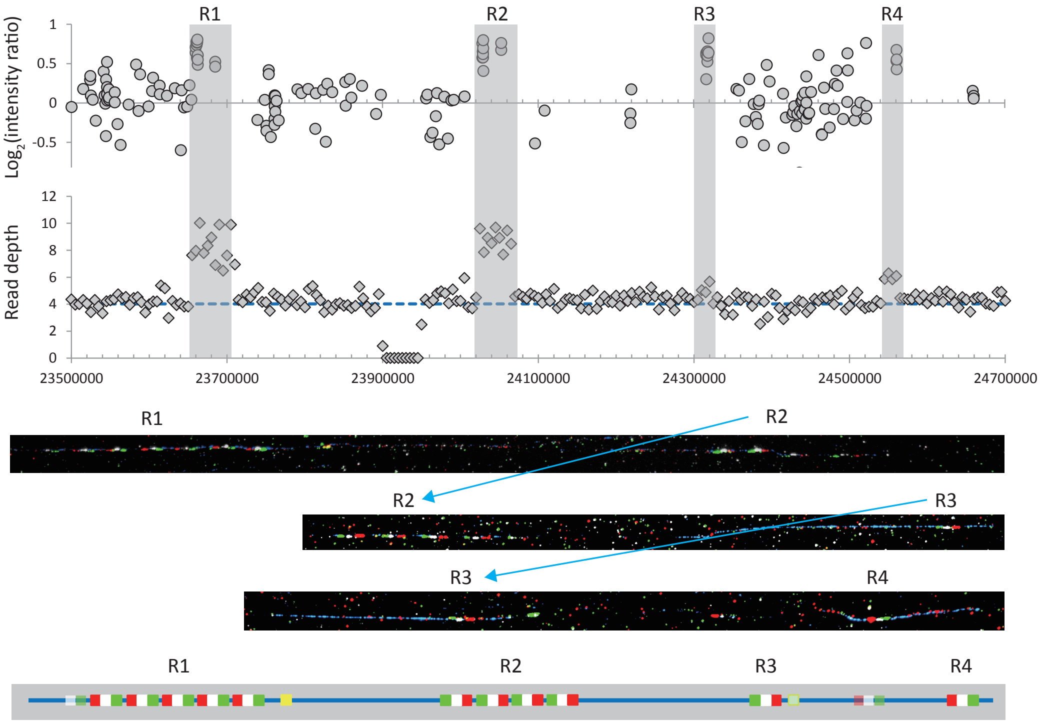

F

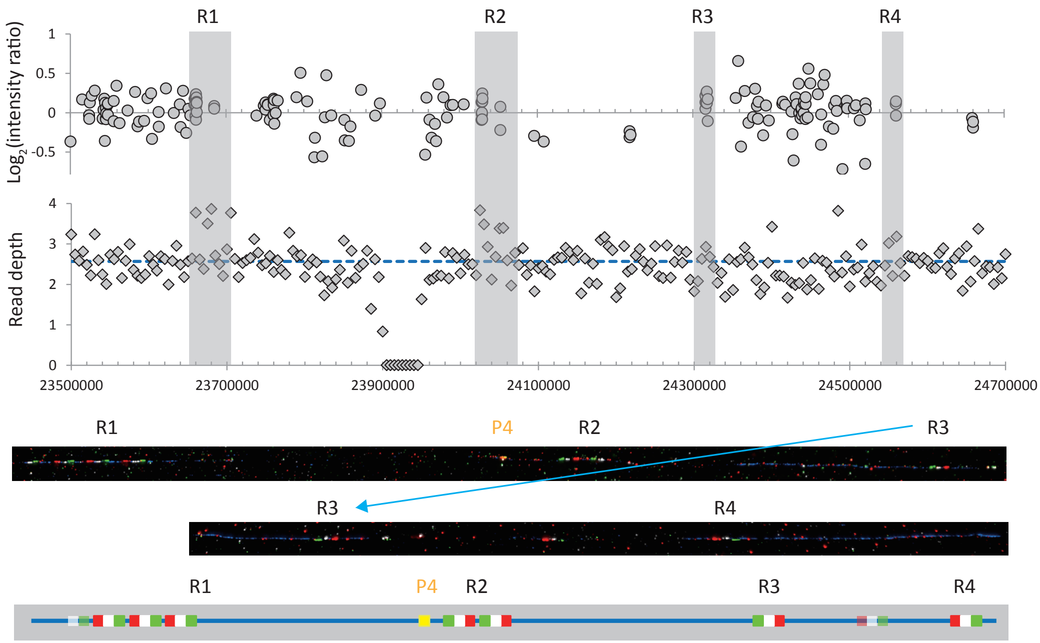

Shi et al Supplementary Figure 1

G

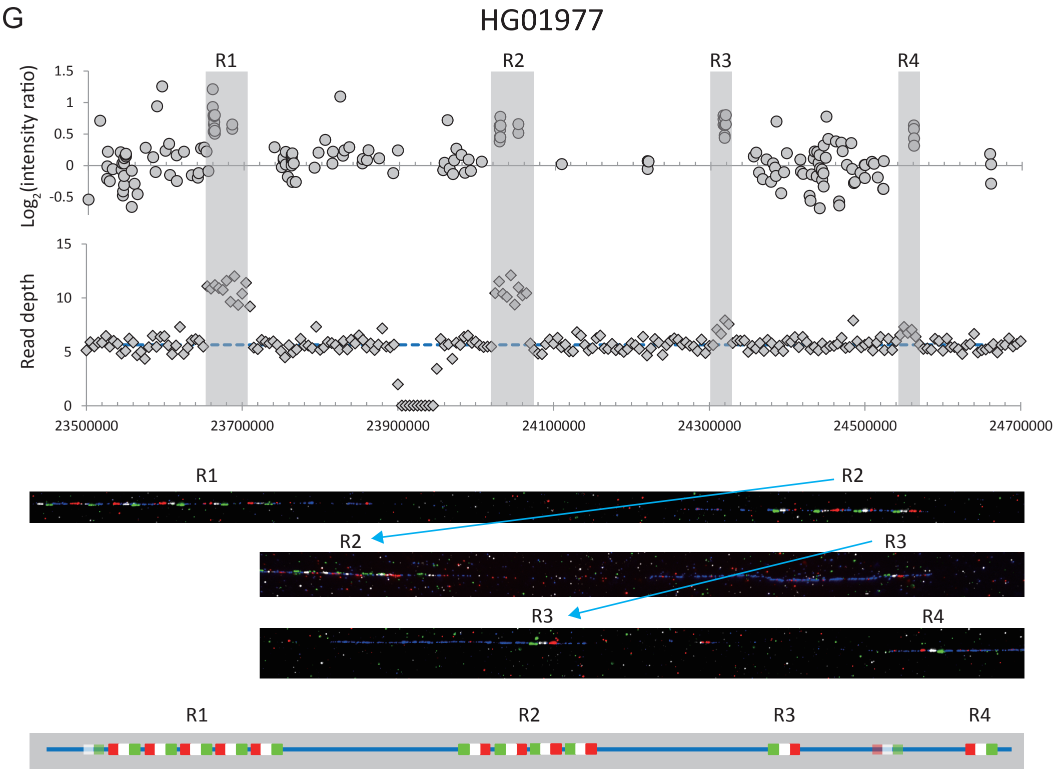

H

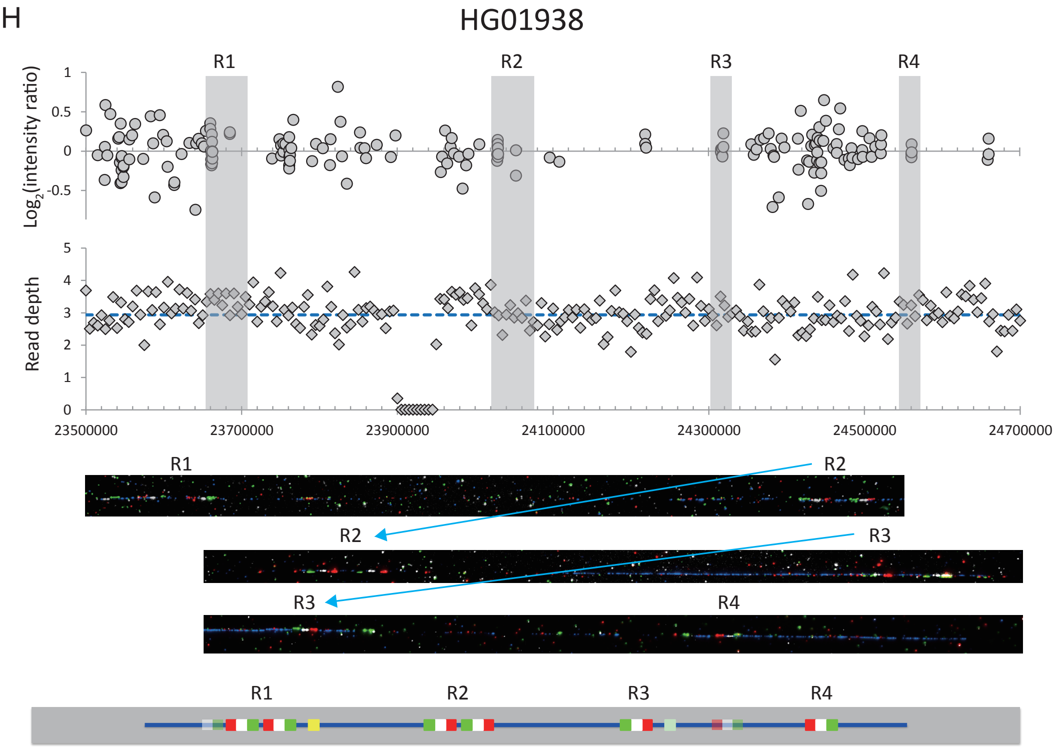

Shi et al Supplementary Figure 1

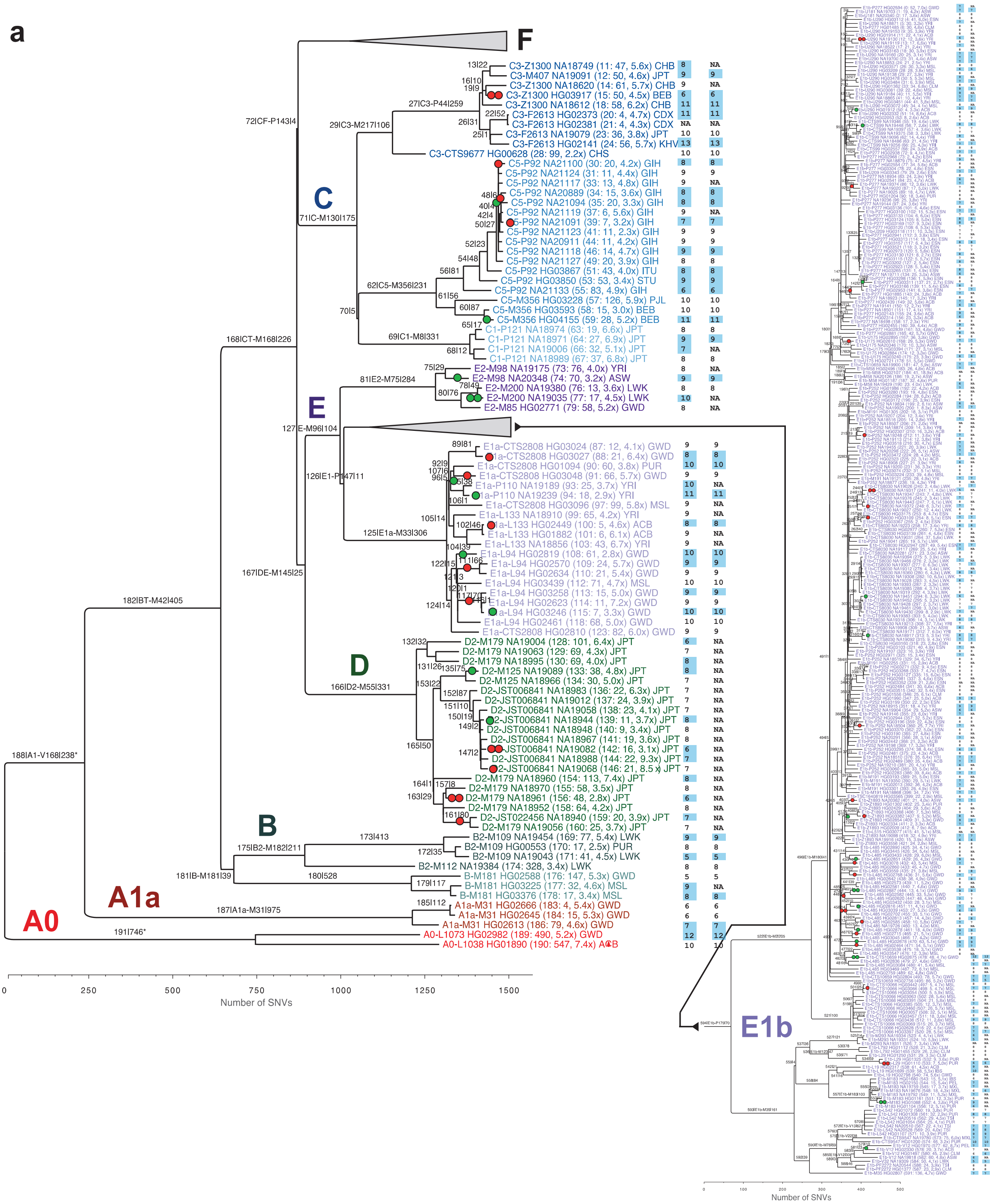

Shi et al Supplementary Figure 2

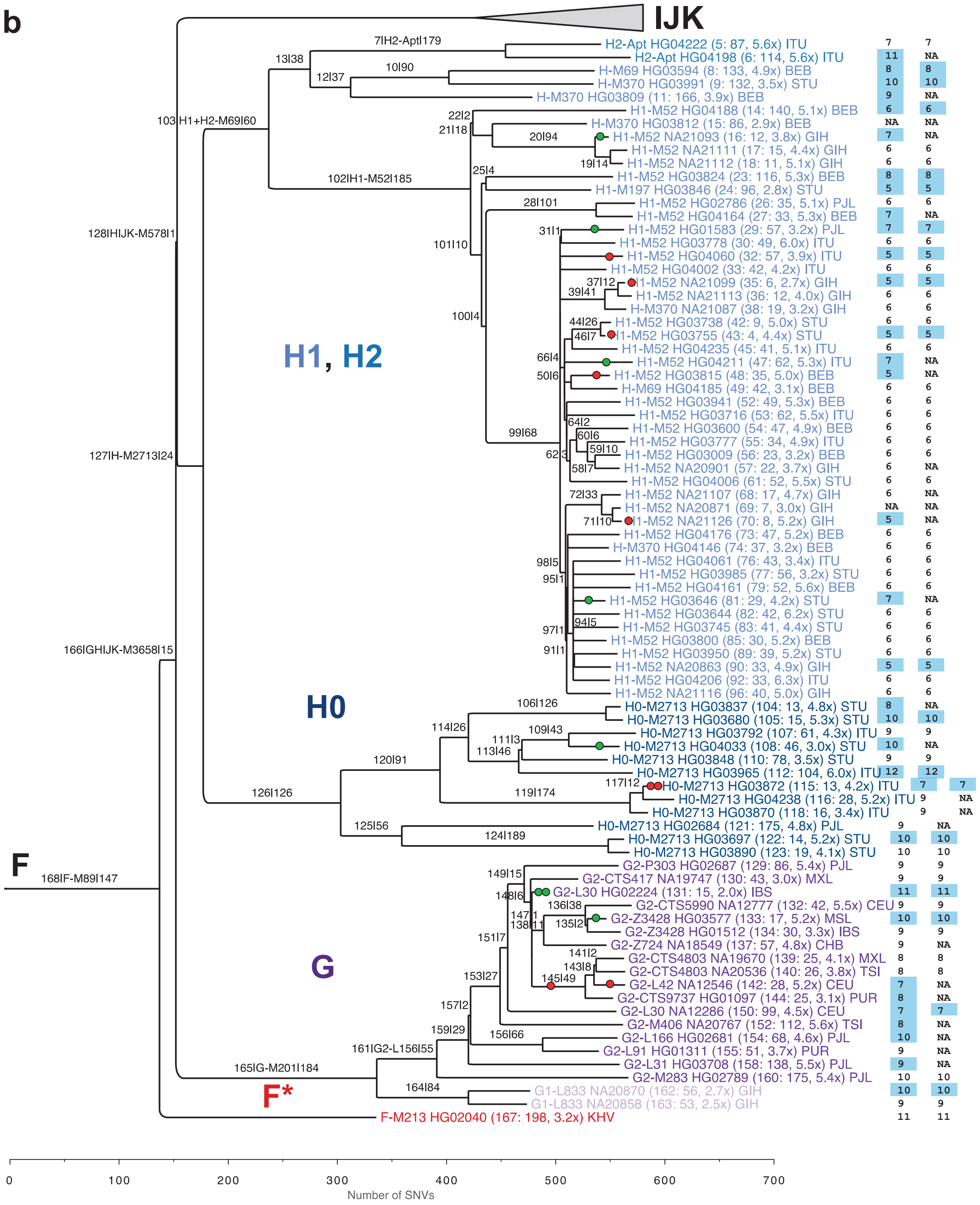

Shi et al Supplementary Figure 2

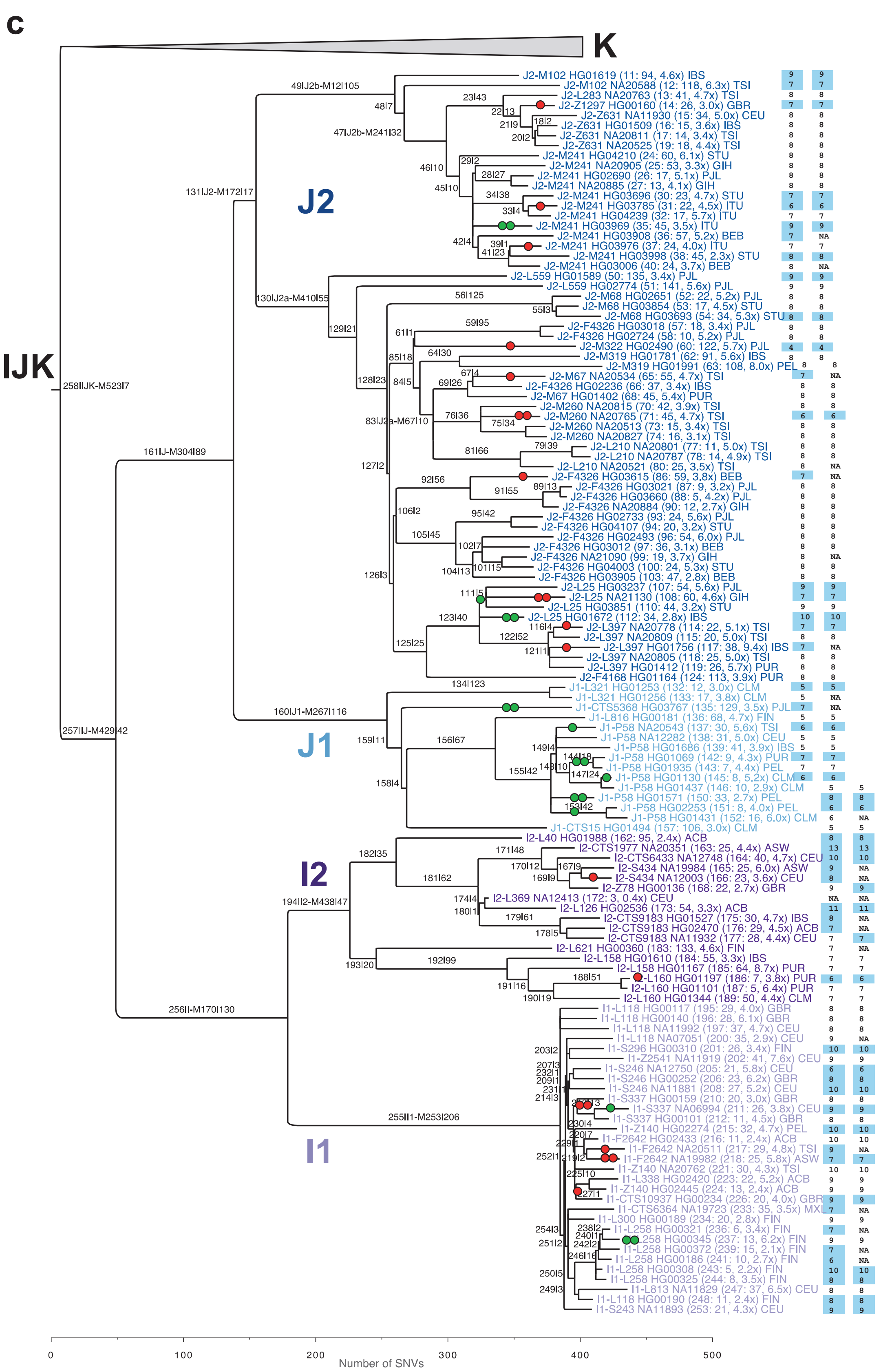

Shi et al Supplementary Figure 2

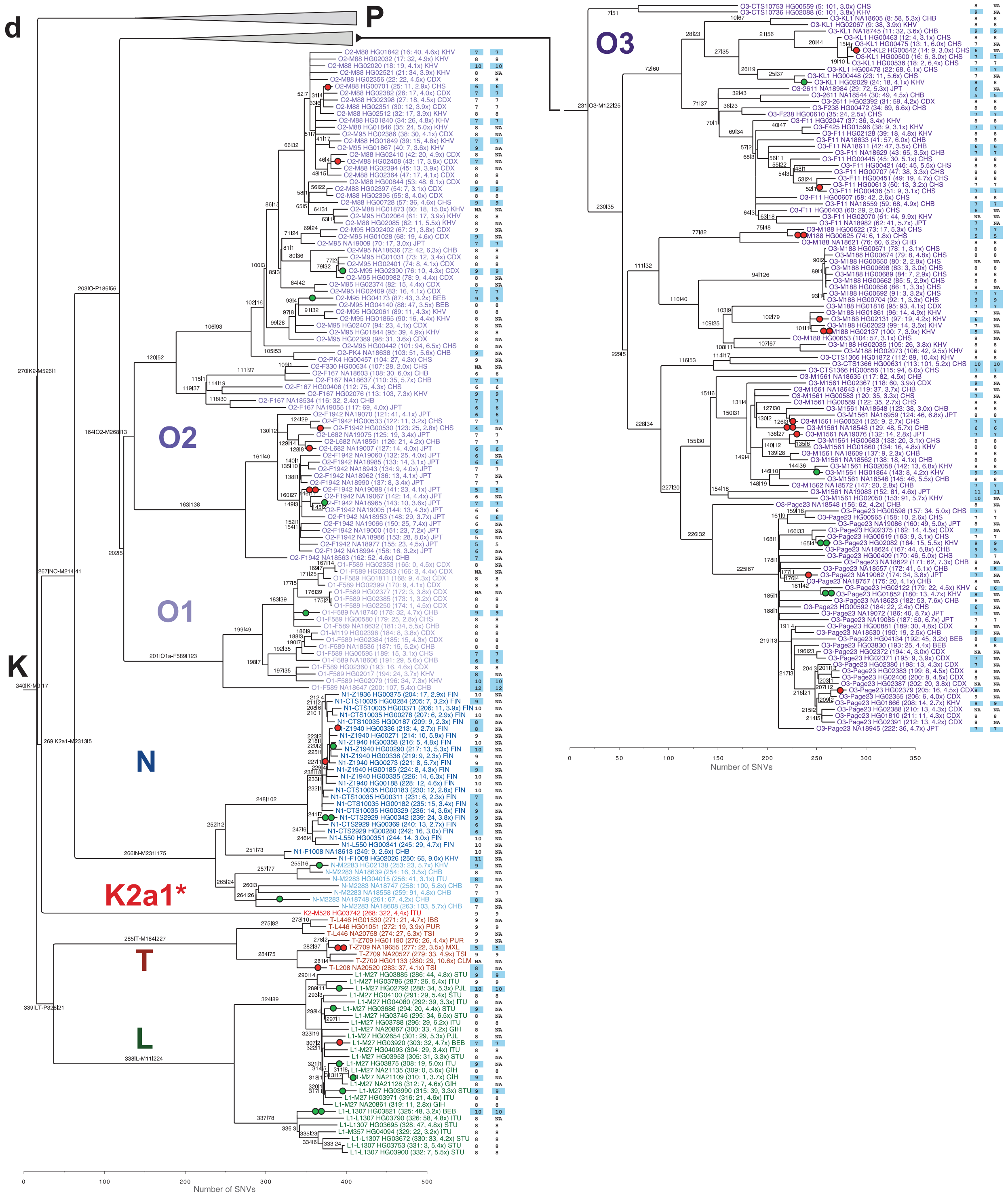

Shi et al Supplementary Figure 2

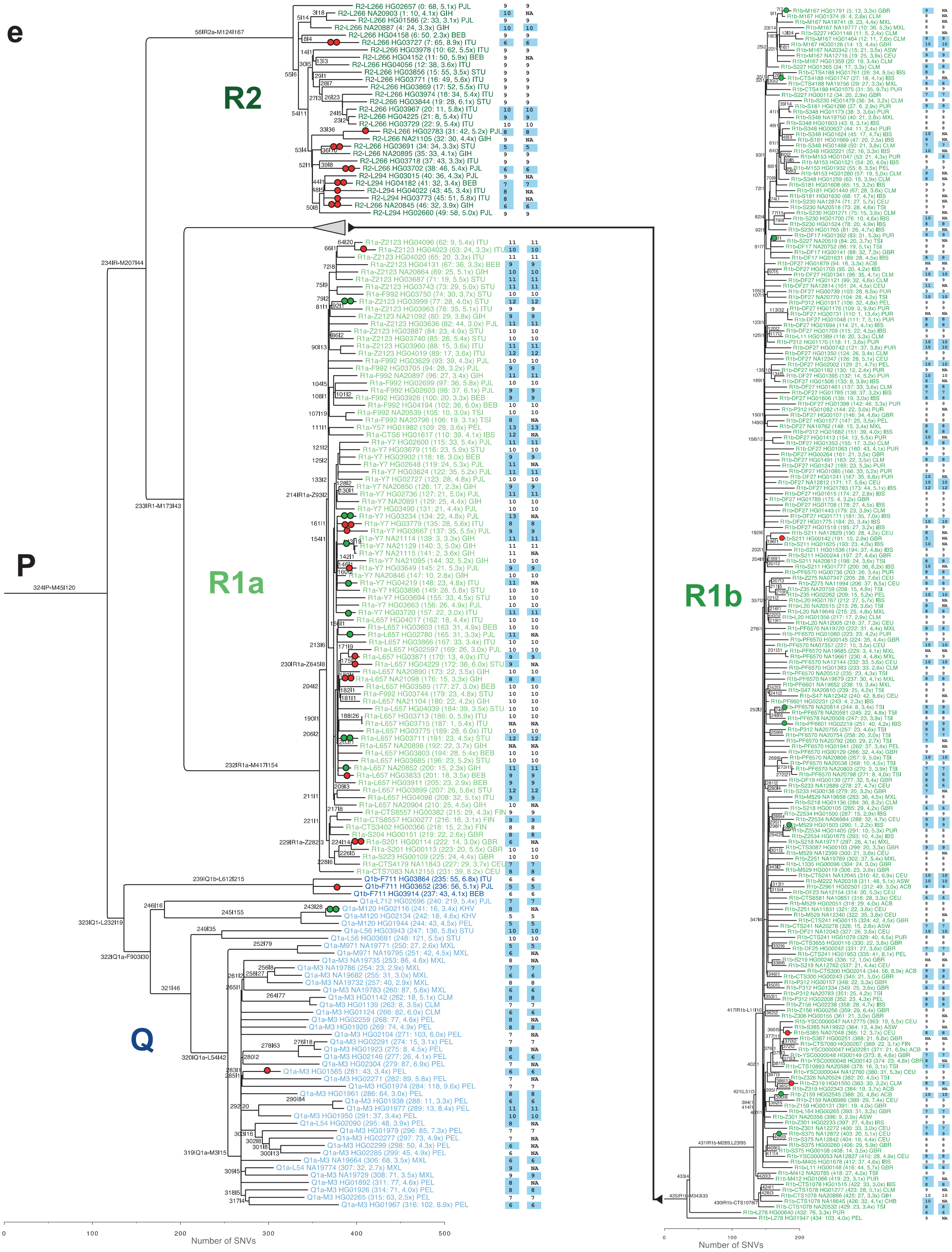

Shi et al Supplementary Figure 2

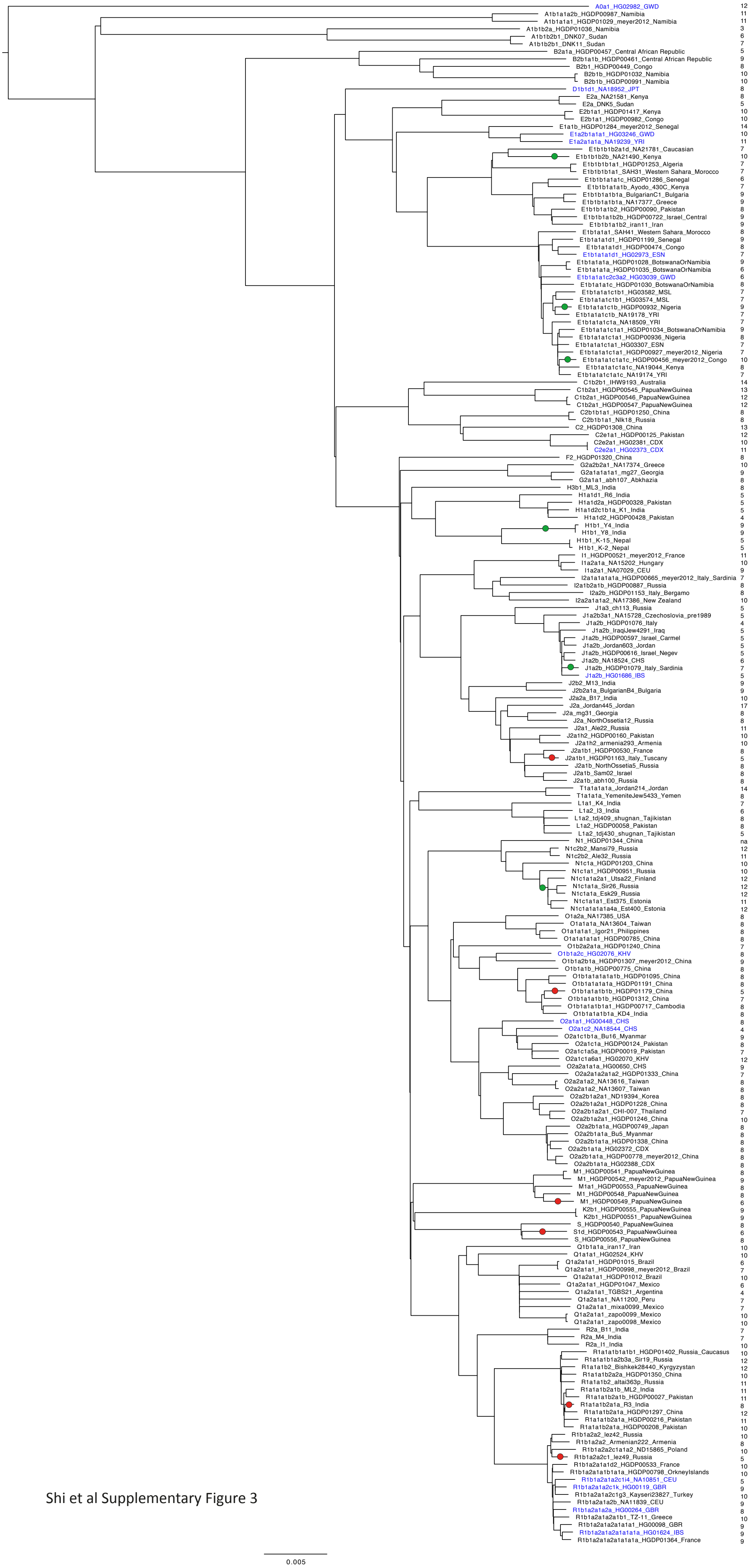

Shi et al Supplementary Figure 3

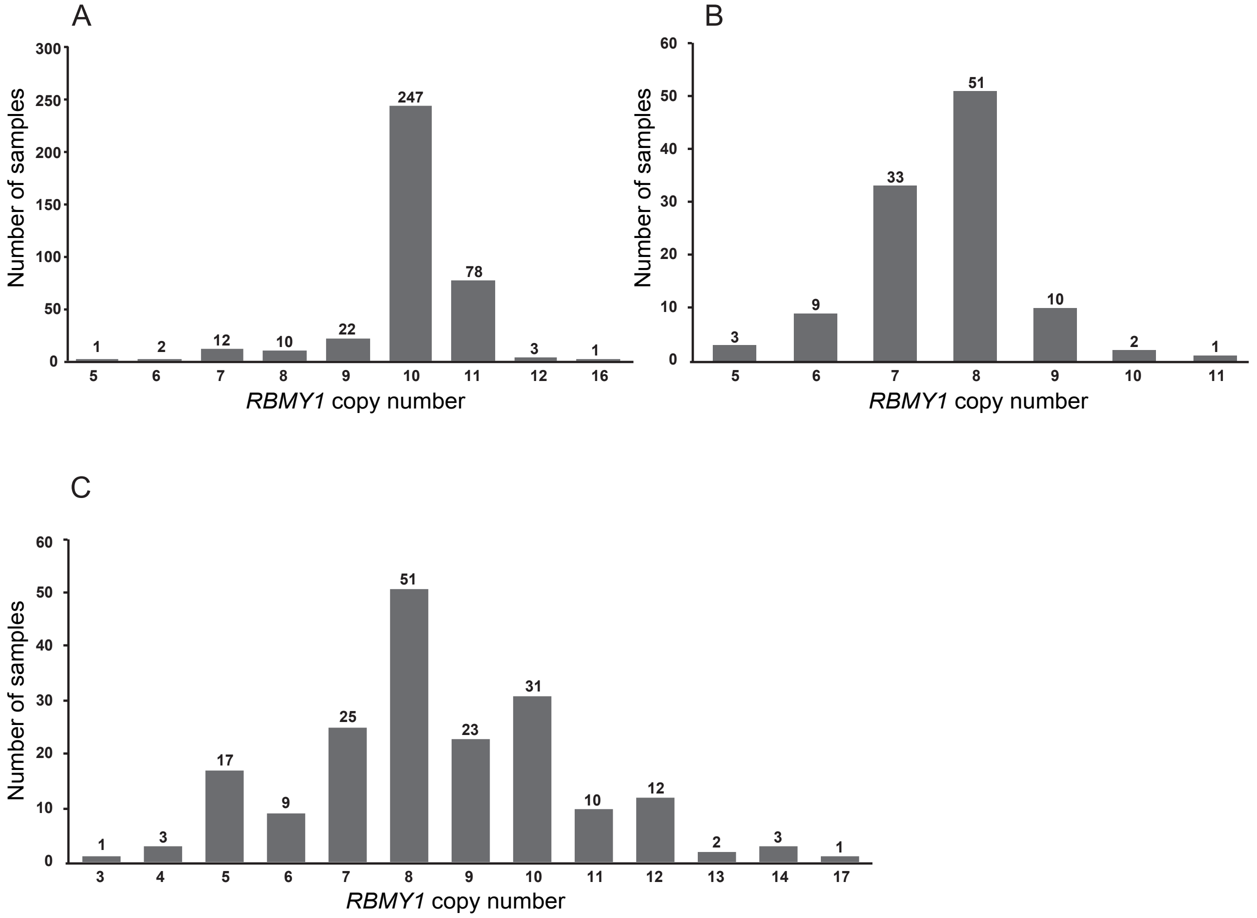

Shi et al Supplementary Figure 4

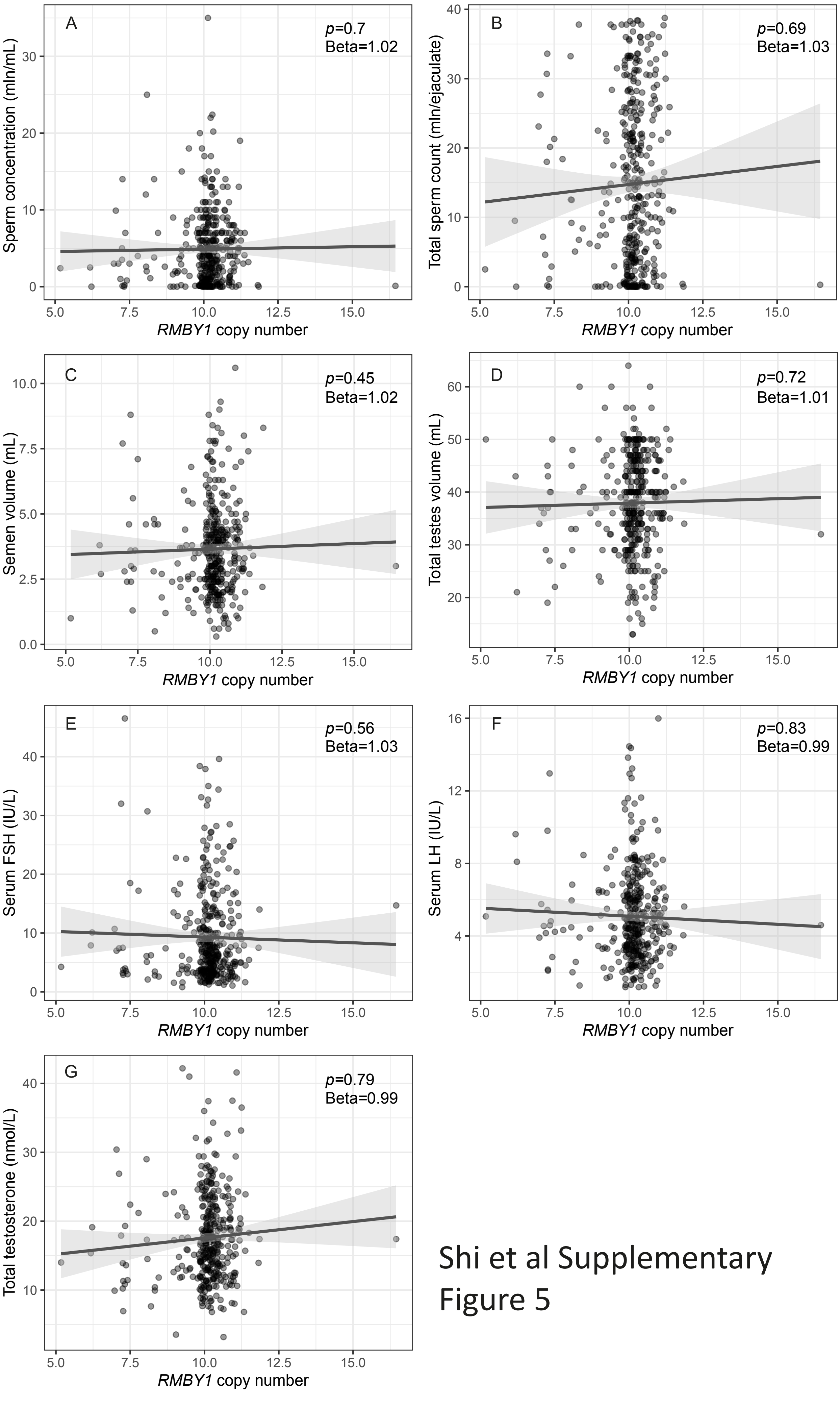

Shi et al Supplementary Figure 5

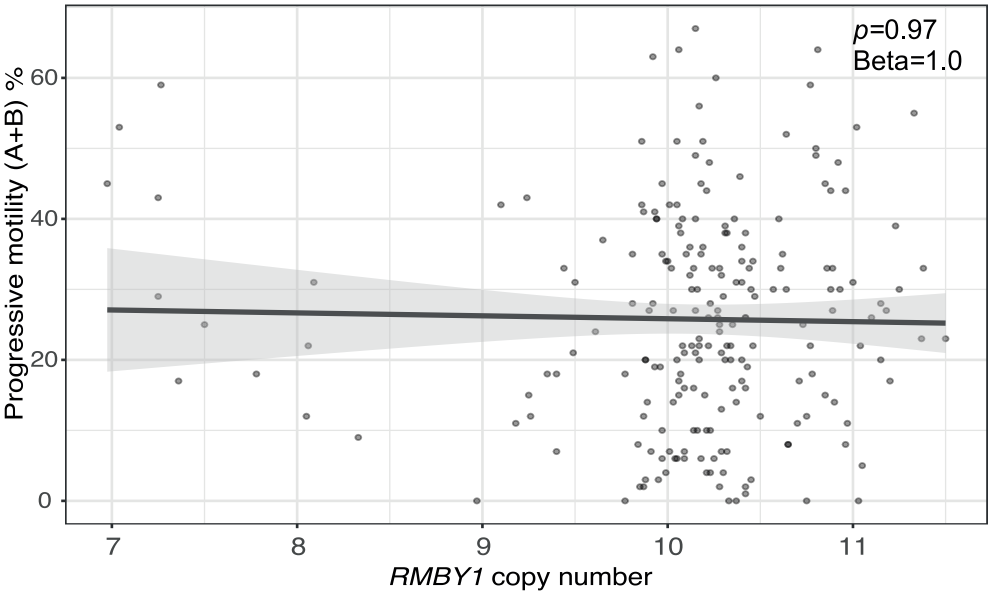

Shi et al Supplementary Figure 6
